# Deciphering the Acylation Pattern of *Yersinia enterocolitica* Lipid A

**DOI:** 10.1371/journal.ppat.1002978

**Published:** 2012-10-25

**Authors:** Mar Reinés, Enrique Llobet, Käthe M. Dahlström, Camino Pérez-Gutiérrez, Catalina M. Llompart, Nuria Torrecabota, Tiina A. Salminen, José A. Bengoechea

**Affiliations:** 1 Laboratory Microbial Pathogenesis, Fundació d'Investigació Sanitària de les Illes Balears (FISIB), Recinto Hospital Joan March, Bunyola, Spain; 2 Consejo Superior de Investigaciones Científicas (CSIC), Madrid, Spain; 3 Structural Bioinformatics Laboratory, Department of Biosciences, Åbo Akademi University, Turku, Finland; Tufts University School of Medicine, United States of America

## Abstract

Pathogenic bacteria may modify their surface to evade the host innate immune response. *Yersinia enterocolitica* modulates its lipopolysaccharide (LPS) lipid A structure, and the key regulatory signal is temperature. At 21°C, lipid A is hexa-acylated and may be modified with aminoarabinose or palmitate. At 37°C, *Y. enterocolitica* expresses a tetra-acylated lipid A consistent with the 3′-O-deacylation of the molecule. In this work, by combining genetic and mass spectrometric analysis, we establish that *Y. enterocolitica* encodes a lipid A deacylase, LpxR, responsible for the lipid A structure observed at 37°C. Western blot analyses indicate that LpxR exhibits latency at 21°C, deacylation of lipid A is not observed despite the expression of LpxR in the membrane. Aminoarabinose-modified lipid A is involved in the latency. 3-D modelling, docking and site-directed mutagenesis experiments showed that LpxR D31 reduces the active site cavity volume so that aminoarabinose containing Kdo_2_-lipid A cannot be accommodated and, therefore, not deacylated. Our data revealed that the expression of *lpxR* is negatively controlled by RovA and PhoPQ which are necessary for the lipid A modification with aminoarabinose. Next, we investigated the role of lipid A structural plasticity conferred by LpxR on the expression/function of *Y. enterocolitica* virulence factors. We present evidence that motility and invasion of eukaryotic cells were reduced in the *lpxR* mutant grown at 21°C. Mechanistically, our data revealed that the expressions of *flhDC* and *rovA*, regulators controlling the flagellar regulon and invasin respectively, were down-regulated in the mutant. In contrast, the levels of the virulence plasmid (pYV)-encoded virulence factors Yops and YadA were not affected in the *lpxR* mutant. Finally, we establish that the low inflammatory response associated to *Y. enterocolitica* infections is the sum of the anti-inflammatory action exerted by pYV-encoded YopP and the reduced activation of the LPS receptor by a LpxR-dependent deacylated LPS.

## Author Summary

Lipopolysaccharide (LPS) is one of the major surface components of Gram-negative bacteria. The LPS contains a molecular pattern recognized by the innate immune system. Not surprisingly, the modification of the LPS pattern is a virulence strategy of several pathogens to evade the innate immune system. *Yersinia enterocolitica* causes food-borne infections in animals and humans (yersiniosis). Temperature regulates most, if not all, virulence factors of yersiniae including the structure of the LPS lipid A. At 21°C, lipid A is mainly hexa-acylated and may be modified with aminoarabinose or palmitate. In contrast, at 37°C, *Y. enterocolitica* expresses a unique tetra-acylated lipid A. In this work, we establish that *Y. enterocolitica* encodes a lipid A deacylase, LpxR, responsible for the lipid A structure expressed by the pathogen at 37°C, the host temperature. Our findings also revealed that the low inflammatory response associated to *Y. enterocolitica* infections is the sum of the anti-inflammatory action exerted by a *Yersinia* protein translocated into the cytosol of macrophages and the reduced activation of the LPS receptor complex due to the expression of a LpxR-dependent deacylated LPS.

## Introduction

Lipopolysaccharide (LPS) is one of the major surface components of Gram-negative bacteria. The molecular structure of LPS is rather unique: an amphiphile with a hydrophobic region, the so-called lipid A, adjacent to a densely negatively charged polysaccharide. In *Escherichia coli* K-12, the lipid A is a β(1′-6)-linked disaccharide of glucosamine phosphorylated at the 1 and 4′ positions with positions 2, 3, 2′, and 3′acylated with *R*-3-hydroxymyristoyl groups, the so-called lipid IV_A_. The 2′and 3′*R*-3-hydroxymyristoyl groups are further acylated with laureate (C_12_) and myristate (C_14_), respectively, by the action of the so-called late acyltransferases LpxL (HtrB) and LpxM (MsbB), respectively [1]. When *E. coli* is grown at 12°C, LpxP, the cold-temperature-specific late acyltransferase, acts instead of LpxL adding palmitoleate (C_16∶1_) [1]. Although the enzymes required to synthesize the lipid A are conserved throughout all Gram-negative bacteria there is heterogeneity on lipid A structure among Gram-negative bacteria compared to the *E. coli* K-12. This is due to differences in the type and length of fatty acids, in the presence of decorations such as aminoarabinose or phosphoethanolamine and even in the removal of groups such as phosphates or fatty acids from lipid A [2].

LPS plays a crucial role during recognition of microbial infection by the host immune system. In fact, the lipid A moiety is a ligand of the Toll-like receptor 4 (TLR4)/myeloid differentiation factor 2 complex [3]. The stimulation of this receptor complex triggers the activation of signalling cascades resulting in the induction of antimicrobial genes and release of cytokines, thereby initiating inflammatory and immune defence responses. Perusal of the literature demonstrates that changes in the number of acyl chains and in the phosphorylation status of the headgroup greatly affect the biological activity of lipid A. It is not surprising that some pathogens modulate their lipid A structure to alter their detection by the host; being these regulated changes important virulence traits (for a review see [4]). Furthermore, given the importance of the LPS structure to the homeostasis of the outer membrane, it is possible that the aforementioned changes may also affect the physiology of the outer membrane as was recently demonstrated for *Salmonella* [5].

The genus *Yersinia* includes three human pathogens: *Y. pestis*, *Y. pseudotuberculosis* and *Y. enterocolitica*. The latter can cause food-borne infections in animals and humans (yersiniosis), with symptoms such as enteritis and mesenteric lymphadenitis [6]. *Y. enterocolitica* is endowed with a repertoire of virulence factors that help bacteria to colonize the intestinal tract and to resist host defence mechanisms [7], [8]. Temperature regulates most, if not all, virulence factors of yersiniae [7], [8]. Recent studies have shown that temperature also regulates the structure of yersiniae lipid A [9]–[14]. Thus the number and type of the lipid A fatty acids and the substitutions of the 1- and 4′-positions in the glucosamine disaccharide can vary. Rebeil and co-workers [12] elegantly demonstrated that a shift in temperature induces a change in the number and type of acyl groups on the lipid A of the three *Yersinia* species. At 21°C, lipid As are mainly hexa-acylated whereas at 37°C they are tetra-acylated [12]. The temperature-dependent regulation of the lipid A acyltransferases underlines the shift in lipid A acylation both in *Y. pestis* and in *Y. enterocolitica* [12], [14]. Pathogenic yersiniae also express hepta-acylated lipid A due to the addition of C_10_, in *Y. pestis* and *Y. pseudotuberculosis*, or C_16_ (palmitate), in *Y. enterocolitica* [12], [14], [15]. PagP is the acyltransferase responsible for the addition of palmitate to the lipid A in *Y. enterocolitica* [15]. Other lipid A species are consistent with the substitution of the phosphate at the 4′ end of the glucosamine disaccharide with aminoarabinose [15]. The aminoarabinose content is temperature-regulated in *Y. pestis* and in *Y. enterocolitica* [12], [15], [16]; being higher in bacteria gown at 21°C than at 37°C. Similar to other Gram-negative bacteria, the products of *ugd* and *pmrHFIJKLM (arnBCADTEF)*(hereafter *pmrF* operon) are required for the synthesis and addition of aminoarabinose to lipid A in *Y. enterocolitica* [15]. Finally, we and others [9]–[14], [17] have reported a unique tetra-acyl lipid A species (*m/z* 1388) found only in *Y. enterocolitica* grown at 37°C. Evidence support the notion that this species lacks the ester-linked *R*-3-hydroxymyristoyl group further acylated with laureate (C_12_) [12], [14], [17]. Indeed, mass spectrometry analysis did confirm that the nonreducing glucosamine of the lipid A is substituted with only one (amide-linked) *R*-3-hydroxymyristoyl group further acylated with myristate (C_14_) [17]. Altogether, these findings strongly suggest that the tetra-acyl lipid A species (*m/z* 1388) may be caused by a deacylase removing the 3′-acyloxyacyl residue of the lipid A. The work described in this article gives experimental support to this hypothesis and explores the impact of the lipid A structure on *Y. enterocolitica* virulence traits.

## Results

### Identification of the *Y. enterocolitica* 3′-O-deacylase of lipid A

**Figure 1:**
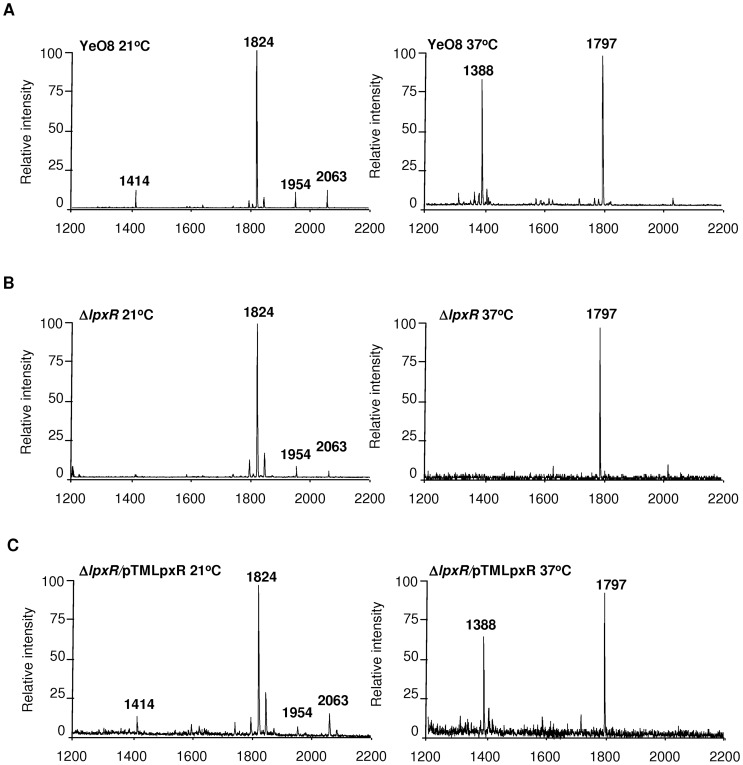
Lipid A analysis from *Y. enterocolitica lpxR* mutant. (A) Negative ion MALDI-TOF mass spectrometry spectra of lipid A isolated from YeO8 grown at 21°C and 37°C. (B) Negative ion MALDI-TOF mass spectrometry spectra of lipid A isolated from YeO8-Δ*lpxR*Km (Δ*lpxR*) grown at 21°C and 37°C. (C) Negative ion MALDI-TOF mass spectrometry spectra of lipid A isolated from YeO8-Δ*lpxR*Km carrying pTMLpxR grown at 21°C and 37°C. The results in all panels are representative of three independent lipid A extractions.

Further confirming previous findings [14], [15], lipid A isolated from *Y. enterocolitica* 8081 serotype O:8 (hereafter YeO8; Table 1) grown at 37°C appeared to be identical to those reported by Rebeil *et al.* and Oertelt *et al.* [12], [17]. The main species were a 3′-O-deacylated form (*m/z* 1388) containing two glucosamines, two phosphates, three 3-OH-C_14_, and one C_14_; and a hexa-acylated form (*m/z* 1797) (Figure 1A). In bacteria grown at 21°C, a minor species (*m/z* 1414) was detected and may represent a 3′-O-deacylated form containing three 3-OH-C_14_ and one C_16∶1_ [12]. *S. enterica* serovar *typhimurium* and *Helicobacter pylori* also express 3′-O-deacylated lipid A species [18], [19]. A membrane located hydrolase, named LpxR, removes the 3′-acyloxyacyl residue of lipid A in both organisms [18], [19]. *In silico* analysis of the YeO8 genome (accession number AM286415; [20]) revealed that this pathogen may encode an LpxR orthologue (locus tag YE3039). The predicted YeO8 LpxR (YeLpxR) has 73% and 20% amino acid identities to *S. enterica* and *H. pylori* LpxR proteins, respectively. Furthermore, YeLpxR has 100% amino acid identity to *Y. enterocolitca* Y11 serotype O:3 (locus tag Y11_05741; accession number FR729477) and Y105 serotype O:9 (locus tag YE105_C2442; accession number CP002246) LpxR homologs. Analysis of the available *Y. pestis* and *Y. pseudotuberculosis* genomes revealed that they do not encode any gene similar to *lpxR*.

**Table 1:** Strains and plasmids used in this study.

Bacterial strains and plasmids	Genotype or comments	Source or reference
**Strains**		
*Escherichia coli*		
C600	*thi thr leuB tonA lacY supE*	[80]
CC118-λ*pir*	Δ(*ara-leu*)7697 *araD139* Δ*lacX74 galE galK* Δ*phoA20 thi-1 rpsE rrpoB argE*(Am) *recA1*	
DH5α-λ*pir*	*DlacU*169 (F80lacZDM15), *recA*1, *endA*1, *hsdR*17, *thi*-1, *gyrA*96, *relA*1, λpir phage lysogen.	
MG1655	*F- lambda- ilvG- rfb-50 rph-1*	
*Yersinia enterocolitica*		
8081-R^−^M^+^ (YeO8)	Derivative of wild type strain 8081; pYV^+^	[81]
8081-c R^−^M^+^ (YeO8c)	R^−^M^+^ derivative of 8081-c the pYV-cured derivative of 8081	[81]
YeO8-Δ*pmrF*	YeO8, Δ*pmrF*, *pmrF* internal fragment deleted by double crossover	[15]
YeO8-Δ*pagP*GB	YeO8, Δ*pagP*::Km-GenBlock, Km^R^, *pagP* gene is inactivated	[15]
YeO8-Δ*lpxR*Km	YeO8, Δ*lpxR*::Km, Km^R^, *lpxR* gene is inactivated	This study
YeO8-Δ*lpxR*	YeO8-Δ*lpxR*Km, Δ*lpxR*, Km cassette removed by Flp-mediated recombination, *lpxR* internal fragment deleted.	This study
YeO8-Δ*pmrF-*Δ*lpxR*KM	YeO8-Δ*pmrF*, Δ*lpxR*::Km, Km^R^, *lpxR* gene is inactivated	This study
YeO8-Δ*pmrAB*	YeO8, Δ*pmrAB*, internal fragment deleted by double crossover	[15]
YeO8-Δ*phoPQ*	YeO8, Δ*phoPQ*, internal fragment deleted by double crossover	[15]
Yvm927	*rovA* deletion mutant in JB580v	[23]
YeO8-Δ*phoPQ*-Δ*pmrAB*	YeO8-Δ*phoPQ*, Δ*pmrAB*; internal fragment deleted by double crossover	[15]
Yvm927-Δ*phoPQ*	Yvm927, Δ*phoPQ* internal fragment deleted by double crossover	[15]
Yvm927-Δ*phoPQ*-Δ*pmrAB*	Yvm927-Δ*phoPQ*, Δ*pmrAB*; internal fragment deleted by double crossover	[15]
YeO8-Δ*yopE*	YeO8, Δ*yopE*, *yopE* internal fragment deleted by double crossover.	This study
YeO8-Δ*yopP*Km	YeO8, Δ*yopP::*Km, KmR, *yopP* gene inactivated	This study
YeO8-Δ*yopP*	YeO8-Δ*yopP*Km, Δ*yopP*, Km cassette removed by Flp-mediated recombination, *yopP* internal fragment deleted.	This study
YeO8-Δ*yopP*-Δ*lpxR*Km	YeO8-Δ*yopP*, Δ*lpxR*::Km, Km^R^, *lpxR* gene is inactivated	This study
**Plasmids**		
pGEM-T Easy	Cloning plasmid, Amp^R^	Promega
p34S-Tp	Source of Tp cassette, Amp^R^, Tp^R^	[64]
pGPL01Tp	Firefly luciferase transcriptional fusion suicide vector, Tp^R^	[15]
pGEMTFRTKm	Km cassette source for mutagenesis flanked by BamHI-FRT sites, Km^R^, Amp^R^	[82]
pFLP2	Plasmid encoding FLP to remove casettes between FRT sites. Mobilizable. *sacB* for counterselection, Amp^R^	[63]
pFLP2Tp	Trimethoprim resistance cassette cloned into ScaI site of pFLP2, Tp^R^	This study
pKNG101	oriR6K Mob^+^, *sacB* for counterselection, Str^R^	[62]
pTM100	Mob^+^, derivated of pACYC184, Cm^R^ Tet^R^	[40]
pTMLpxR	1.5 kb wild-type *lpxR* locus cloned into pTM100, Tet^R^	This study
pTMLpxRFLAG	1.4 kb wild-type locus with a *flag* sequence cloned into pTM100, Tet^R^	This study
pTMLpxR(N9A)FLAG	1.4 kb *lpxR* allele with a *flag* sequence cloned into pTM100, Tet^R^	This study
pTMLpxR(S34A)FLAG	1.4 kb *lpxR* allele with a *flag* sequence cloned into pTM100, Tet^R^	This study
pGEMTΔ*lpxR*	pGEM-T Easy containing Δ*lpxR* deleted gene, Amp^R^	This study
pGEMTΔ*lpxR*Km	pGEM-T Easy containing Δ*lpxR*::Km, Km^R^, Amp^R^	This study
pGEMTΔ*yopE*	pGEM-T Easy containing Δ*yopE* deleted gene, Amp^R^	This study
pGEMTΔ*yopP*	pGEM-T Easy containing Δ*yopP* deleted gene, Amp^R^	This study
pGEMTΔ*yopP*Km	pGEM-T Easy containing Δ*yopP*::Km, Km^R^, Amp^R^	This study
pKNGΔ*lpxR*Km	pKNG101 containing Δ*lpxR*::Km, Km^R^, Str^R^	This study
pKNGΔ*yopP*Km	pKNG101 containing Δ*yopP*::Km, Str^R^,Km^R^	This study
pKNGΔ*YopE*	pKNG101 containing Δ*yopE*, Str^R^	This study
pRVPro*rovA*lucFF	pRV1 containig *rovA*::*lucFF*; Cm^R^	[14]
pGPL01TpYelpxR	pGPL01Tp containing a 443-bp DNA fragment corresponding to the *lpxR* promoter region, Tp^R^	This study
pDHS45	pFUSE containing *yplXA'::lacZYA*, Cm^R^	[29]
pRSFlhDC08	pRV1 containing *flhDC::lucFF*, Cm^R^	[26]
pRVProrovAlucFF	pRV1 containig *rovA::lucFF*, Cm^R^	[14]
pINP41	pEP184 containing *invΔ412::phoA*, Clm^R^	[32]

YeO8 *lpxR* was mutated to determine whether this gene is indeed responsible for removing the 3′-acyloxyacyl residue of lipid A. MALDI-TOF mass spectrometry studies showed that, at 37°C, the *lpxR* mutant (YeO8-Δl*pxR*Km) produced a lipid A which lacked the unique tetra-acyl lipid A species (*m/z* 1388) found in YeO8 and only contained the hexa-acylated species (*m/z* 1797; four 3-OH-C_14_, one C_12_ and one C_14_) (Figure 1B). At 21°C, lipid A isolated from YeO8-Δl*pxR*Km was similar to that of YeO8 although without the minor species *m/z* 1414 (Figure 1B). Complementation of the mutant with pTMLpxR restored the presence of the tetra-acyl species (Figure 1C).

In summary, our results confirmed the predicted function of *Y. enterocolitica* O:8 *lpxR* homolog as the lipid A 3′-O-deacylase.

### Expression of *lpxR*

**Figure 2:**
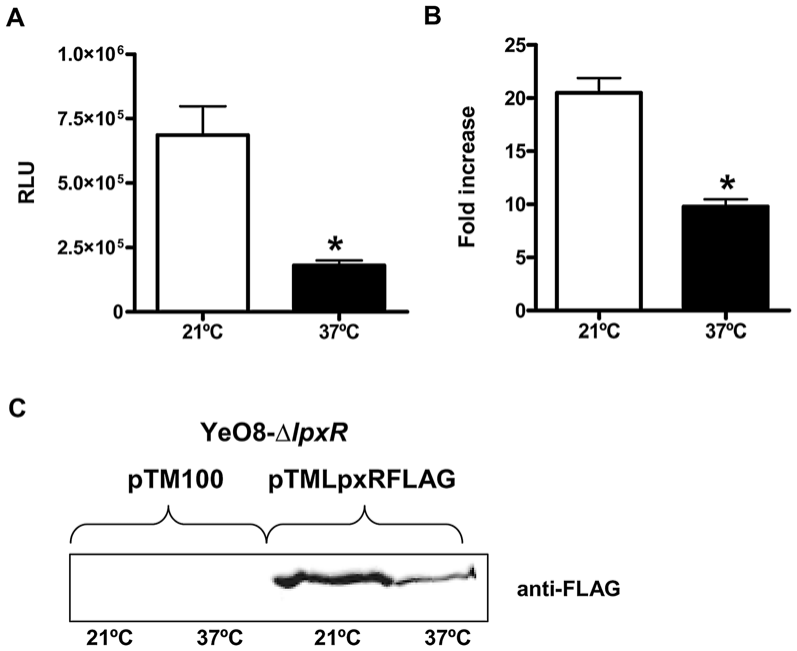
Temperature regulates the expression of *Y. enterocolitica lpxR*. (A) Analysis of the expression of *lpxR* by measuring luciferase activity of YeO8 carrying *lpxR::lucFF* transcriptional fusion, which was grown at 21°C (white bars) or 37°C (black bars). Data are presented as mean ± SD (n = 3). *, results are significantly different (p<0.05; two-tailed *t* test) from the results for bacteria grown at 21°C. (B) Analysis of *lpxR* mRNA levels by RT-qPCR. Total RNA was extracted from bacteria grown at 21°C (white bar) or 37°C (black bar). Data are presented as mean ± SD (n = 3). *, results are significantly different (p<0.05; two-tailed *t* test) from the results for bacteria grown at 21°C. (C) Western blot analysis of LpxR FLAG tagged levels. Cell envelopes were purified from YeO8-Δ*lpxR*Km mutant carrying pTM100 or pTMLpxRFLAG plasmids. 80 µg of proteins were run in SDS-12% polyacrylamide gel, electrotransferred onto a nitrocellulose membrane, and developed by using anti-Flag antibodies.

The LpxR-dependent lipid A deacylation was more evident on bacteria grown at 37°C than at 21°C, hence suggesting that the expression and/or function of the deacylase might be temperature-regulated, being higher at 37°C than at 21°C. To monitor transcription of *lpxR* quantitatively, a transcriptional fusion was constructed in which a promoterless *lucFF* gene was under the control of the *lpxR* promoter region (see Material and Methods); thereafter *lpxR::lucFF* was introduced into YeO8 and the luciferase activity was determined. The expression of the fusion was higher at 21°C than at 37°C (Figure 2A). Real time (RT) quantitative PCR (RT-qPCR) experiments showed that *lpxR* mRNA levels were also higher at 21°C than at 37°C (Figure 2B).

To assess LpxR levels, the C-terminus of the protein was tagged with a FLAG epitope and the construct was cloned into the medium-copy plasmid pTM100 to obtain pTMLpxRFLAG (see Materials and Methods). This plasmid restored the presence of the tetra-acyl species (*m/z* 1414 and *m/z* 1388) in the lipid A of YeO8-Δl*pxR*Km (data not shown). Western blot analysis of purified membranes from YeO8-Δ*lpxR*Km containing pTMLpxRFLAG showed that LpxR levels were higher in membranes from bacteria grown at 21°C than at 37°C (Figure 2C). Altogether, it can be concluded that the expression of *lpxR* is indeed temperature-regulated but, in contrast to our initial hypothesis, its expression is higher at 21°C than at 37°C.

**Figure 3:**
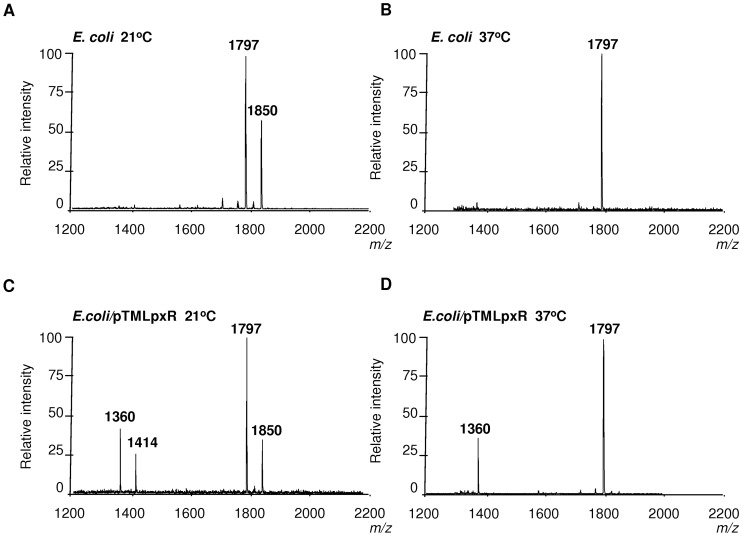
Lipid A analysis from *E. coli* expressing *Y. enterocolitica lpxR*. Negative ion MALDI-TOF mass spectrometry spectra of lipid A isolated from: (A) *E. coli* MG1655 (*E. coli*) grown at 21°C. (B) *E. coli* MG1655 (*E. coli*) grown at 37°C. (C) *E. coli* MG1655 (*E. coli*) carrying pTMLpxR grown at 21°C. (D) *E. coli* MG1655 (*E. coli*) carrying pTMLpxR grown at 37°C. The results in all panels are representative of three independent lipid A extractions.

The apparent contradiction between the mass spectrometry analysis, more deacylation at 37°C, and the Western blot data, higher levels of LpxR at 21°C than at 37°C, led us to explore whether low temperature may affect the function of the enzyme. Since *E. coli* has been used as surrogate host to characterize *Salmonella* LpxR (StLpxR) function [18], we mobilized pTMLpxR into *E. coli* MG1655 to analyze lipid A species by mass spectrometry in bacteria grown at 21°C and 37°C. Results shown in figure 3 demonstrate that LpxR did deacylate the *E. coli* lipid A from bacteria grown either at 21 or 37°C as detected by the presence of species *m/z* 1360 (Figure 3C–D). This species was found previously in *E. coli* expressing StLpxR [18]. Of note, the species *m/z* 1414, which is consistent with the deacylation of the species *m/z* 1850 containing palmitoleate (C_16∶1_) instead of laureate (C_12_), was observed only in *E. coli* grown at 21°C. LpxP is the cold-temperature-specific late acyltransferase responsible for the addition of palmitoleate [1]. Altogether, our results indicate that the reduced LpxR-dependent deacylation found in YeO8 grown at 21°C cannot be attributed to a general lack of function of the enzyme at this temperature.

### Lipid A modification with aminoarabinose affects LpxR-dependent deacylation

**Figure 4:**
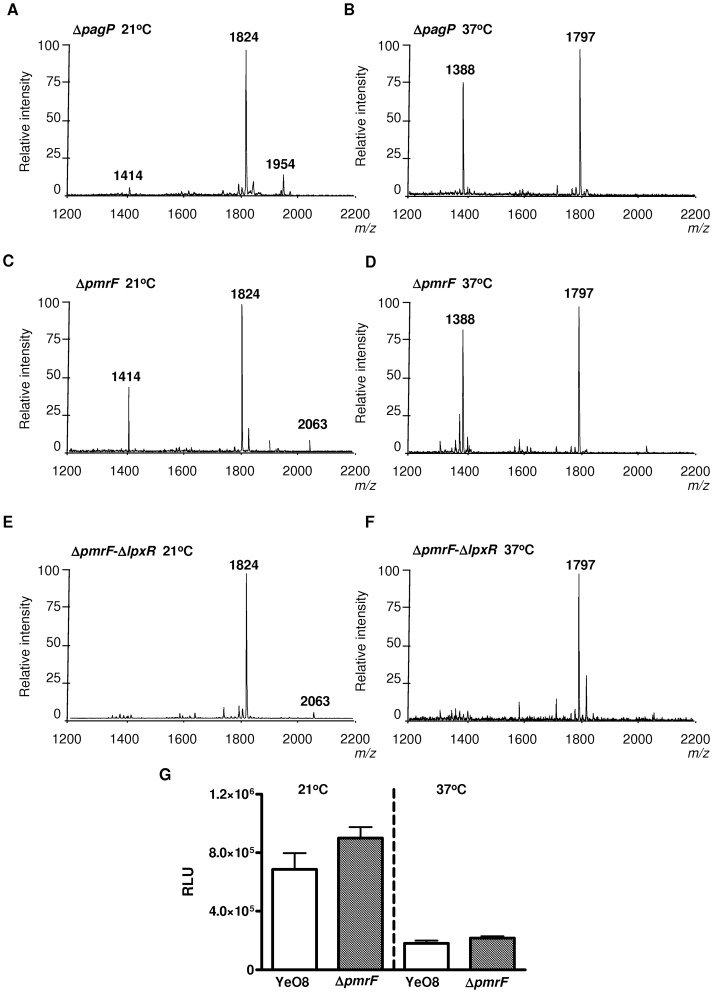
Lipid A analysis from *Y. enterocolitica* lipid A mutants. Negative ion MALDI-TOF mass spectrometry spectra of lipid A isolated from the indicated *Y. enterocolitica* strains grown at 21°C (A,C,E) and 37°C (B,D,F). The results in all panels are representative of three independent lipid A extractions. (G) Analysis of the expression of *lpxR* by measuring luciferase activity of YeO8 (white bars) and YeO8-Δ*pmrF* (gray bars) carrying *lpxR::lucFF* transcriptional fusion, which were grown at 21°C or 37°C. Data are presented as mean ± SD (n = 3).

We sought to determine why LpxR activity was not observed in YeO8 grown at 21°C despite the detection of the enzyme in the membrane. Among other possibilities, we speculated that specific features of YeO8 lipid A found only at 21°C might be responsible for the reduced LpxR activity. Furthermore, these features should be absent in *E. coli* grown at 21°C since LpxR-dependent activity was observed here. A conspicuous difference between YeO8 and *E. coli* lipid As is the presence of aminoarabinose and palmitate (*m/z* 1954 and 2063, respectively) only in the former [14], [15]. Therefore, we explored whether any of these modifications could account for the reduced LpxR activity. In YeO8, similarly to other Gram-negative pathogens, the products of the *pmrF* operon are required for the synthesis and addition of aminoarabinose to lipid A whereas the acyltransferase PagP is required for the addition of palmitate to lipid A [15]. The lipid A from the *pagP* mutant, YeO8-Δ*pagP*GB, grown at 21°C resembled that of the wild-type strain, except that the species containing palmitate (*m/z* 2063) was not detected (Figure 4A). In contrast, the tetra-acylated species (*m/z* 1414) was clearly observed in the lipid A from YeO8-Δ*pmrF* grown at 21°C (Figure 4C). This was dependent on LpxR activity since the peak was absent in the double mutant YeO8-Δ*pmrF-*Δ*lpxR*Km (Figure 4E). LpxR-dependent deacylation of lipid A (*m/z* 1388) observed in bacteria grown at 37°C was not affected in either *pmrF* or *pagP* single mutants (Figure 4B, D). Control experiments revealed that *lpxR* expression was not affected in YeO8-Δ*pmrF* since the expression of the *lpxR::lucFF* fusion was not significantly different between YeO8 and the *pmrF* mutant either grown at 21°C or at 37°C (Figure 4G).

On the whole, these results are consistent with the notion that the reduced LpxR activity observed in YeO8 at 21°C is associated with the lipid A modification with aminoarabinose.

### LpxR 3-D modelling

**Figure 5:**
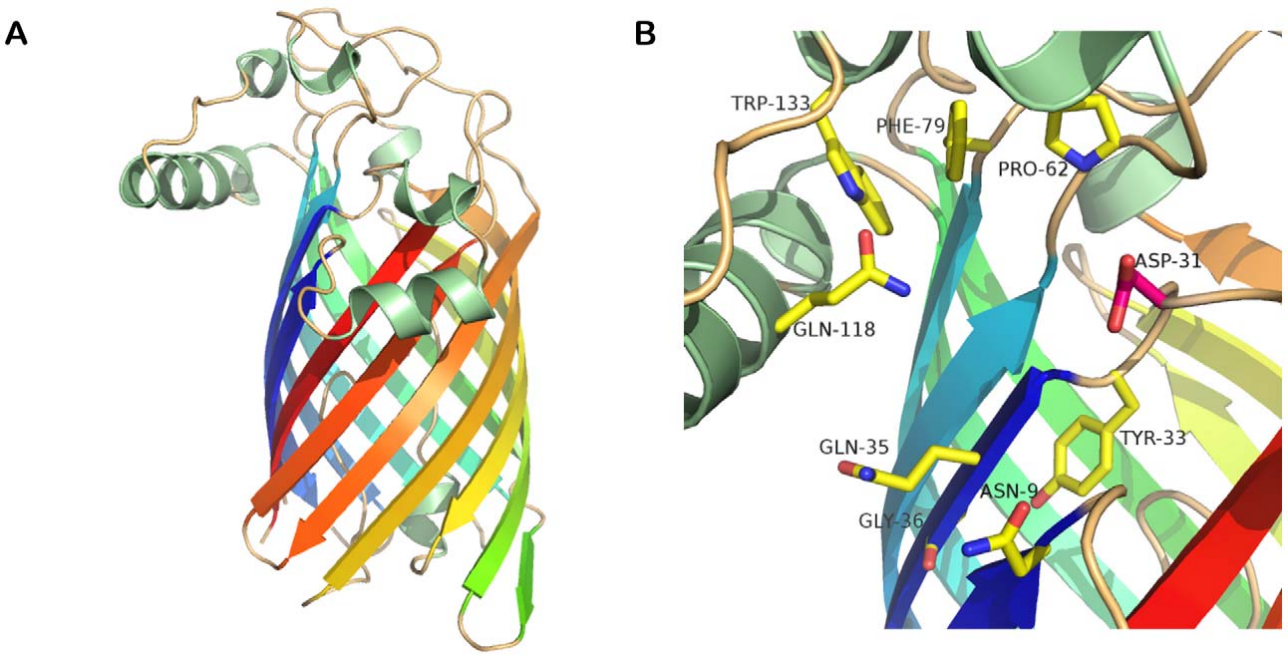
Modelling of *Y. enterocolitica* O:8 LpxR. (A) The model of YeLpxR based on the StLpxR crystal structure. The β-barrel is colored as rainbow, the helices are green and loops are wheat. (B) Close-up view of the active site. The amino acids mutated in this study are shown as sticks in yellow and pink.

**Figure 6:**
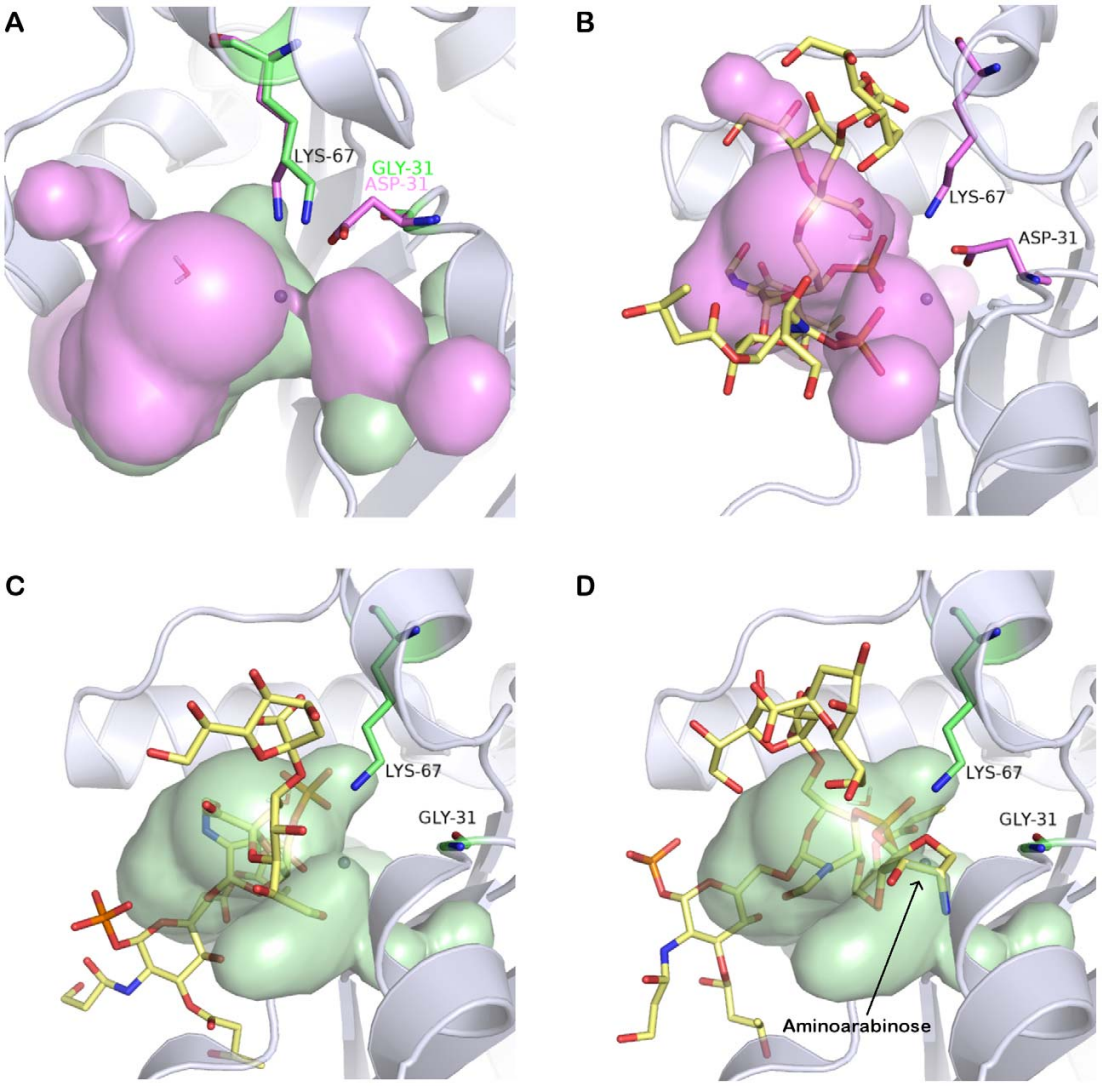
Docking of Kdo_2_-lipid A to LpxR. (A) The differences in surface cavities between YeLpxR (pink) and StLpxR (green) with K67 as sticks with pink carbon atoms for YeLpxR and green carbon atoms for StLpxR and YeLpxR D31 as sticks with pink carbon atoms. G31 in StLpxR is hidden behind D31 in YeLpxR. (B) The YeLpxR model with Kdo_2_-lipid A (sticks with yellow carbon atoms) in the active site. The surface cavity of YeLpxR is shown in pink and K67 and D31 as sticks with pink carbon atoms. (C) The StLpxR model with Kdo_2_-lipid A (yellow sticks) in the active site. The surface cavity of StLpxR is shown in green and K67 and G31 as sticks with green carbon atoms. (D) The StLpxR model with Kdo_2_-lipid A including aminoarabinose in the active site. The surface cavity of StLpxR is shown in green along with K67 and G31 (green sticks).

Our findings might suggest that aminoarabinose-containing LPS may directly inactivate the lipid A deacylase activity of YeLpxR. Alternatively, modification of lipid A with aminoarabinose could inhibit the physical interaction of LPS with YeLpxR. To explore this, the 3-D structure of YeLpxR was modeled (Figure 5A). The amino acids 1–296 (following the putative signal sequence) could be modeled based on the crystal structure of StLpxR (PDB code 3FID; [21]) and the sequence alignment between StLpxR and YeLpxR (Figure S1). The fold of the resulting model is likely to be of good quality, since YeLpxR has such a high sequence identity to StLpxR (75%). Additionally, the important StLpxR amino acids identified by Rutten and co-workers [21] are conserved in YeLpxR. Six amino acids differ between the YeLpxR and the StLpxR active sites (Figure S1). Major differences are D31 and Q35 in YeLpxR, of which D31 is closer to the active site (Figure 5B). The corresponding amino acids are much smaller in StLpxR, glycine and an alanine, respectively, which cause StLpxR to have a bigger cavity. StLpxR has a protruding cavity close to K67, which cannot be found in YeLpxR (Figure 6A). The difference is induced by D31 in YeLpxR, which occupies more space than G31 in StLpxR. As a consequence, the conserved K67 adopts a different conformation in the YeLpxR model. Due to D31, the cavity in YeLpxR is divided into two parts with a narrow connection, and this amino acid also prevents YeLpxR from forming an inward protruding cavity similar to the one found near G31 in StLpxR (Figure 6A).

Docking of a modified Kdo_2_-lipid A molecule (see Materials and methods) to the model of YeLpxR showed that the phosphate group, which attaches aminoarabinose to Kdo_2_-lipid A, binds into the cavity in the vicinity of K67 and D31 (Figure 6B). Docking of the same molecule to the crystal structure of StLpxR yielded a result where the phosphate group was located in the protruding cavity close to K67 (Figure 6C). As expected, docking of the modified Kdo_2_-lipid A molecule with aminoarabinose to the YeLpxR model did not give any valuable result. On the other hand, when the same molecule was docked to the StLpxR crystal structure, aminoarabinose was bound close to G31. It occupies the space corresponding to the narrow connection of the two larger cavities in YeLpxR (Figure 6D)

As a result from the modeling and docking studies, we suggest that Kdo_2_-lipid A with aminoarabinose cannot fit into the active site of YeLpxR due to D31, hence leading to the inability of YeLpxR to deacylate Kdo_2_-lipid A with aminoarabinose.

### *lpxR* site-directed mutagenesis

**Figure 7:**
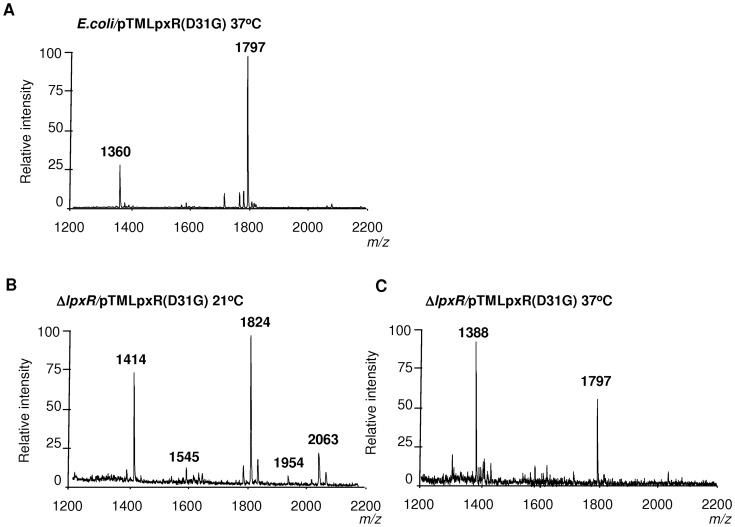
Presence of D31 in the active site pocket of *Y. enterocolitica* O:8 LpxR affects the deacylation activity of the enzyme. Negative ion MALDI-TOF mass spectrometry spectra of lipid A isolated from: (A) *E. coli* MG1655 (*E. coli*) carrying pTMLpxR(D31G) grown at 37°C. (B) YeO8-Δ*lpxR*Km (Δ*lpxR*) carrying pTMLpxR(D31G) grown at 21°C. (C) YeO8-Δ*lpxR*Km (Δ*lpxR*) carrying pTMLpxR(D31G) grown at 37°C. The results in all panels are representative of three independent lipid A extractions.

To confirm our predictions, we constructed LpxR mutants by site-directed mutagenesis (see Material and Methods). In addition to the amino acids corresponding to the active site amino acids in StLpxR, we wanted to study the effect of the D31G mutation for YeLpxR as the modelling and docking studies suggested that D31 has an important role in the YeLpxR specificity for the Kdo_2_-lipid A species. The constructs were introduced into *E. coli* MG1655 and the lipid A from the transformants grown at 37°C was analyzed by MALDI-TOF mass spectrometry. Most of the constructs containing LpxR mutants did trigger the deacylation of *E. coli* lipid A, detected by the presence of species *m/z* 1360, (Table 2). In contrast, constructs containing LpxR mutants, LpxR(N9A), LpxR(D10A), LpxR(S34A), and LpxR(H122A) did not deacylate *E. coli* lipid A. These results were expected since Rutten and co-workers have reported that these residues are located in the StLpxR active site and all of them are conserved in LpxR homologues [21]. Next, only those constructs triggering deacylation of *E. coli* lipid A were introduced into YeO8. When the YeO8 strains were grown at 37°C, all LpxR mutants restored the presence of the tetra-acyl species (*m/z* 1388) in the lipid A of YeO8-Δ*lpxR*Km (Table 2). Additionally, the mass spectrometry analysis revealed that LpxR(D31G) mutant did trigger the deacylation of lipid A in bacteria grown at 21°C as it was detected the presence of lipid A species *m/z* 1414 and *m/z* 1545 (Figure 7B). The latter is consistent with the deacylation of the lipid A species modified with aminoarabinose (*m/z* 1954).

**Table 2:** Effect of *lpxR* mutations on lipid A deacylation.

	Lipid A deacylation	
Mutations	*E. coli* (37°C) *m/z* 1360	*Y. enterocolitica* (21°C) *m/z* 1414
N9A	No	No
D10A	No	n.a.
S34A	No	n.a.
H122A	No	n.a.
Q118A	No	No
Q57A	Yes	No
Y130A	Yes	No
G36A	Yes	No
F79A	Yes	No
P62A	Yes	No
W133A	Yes	No
D31G	Yes	Yes

n.a.; Not analyzed.

In summary, our results further confirmed the amino acids important for the catalytic activity of YeLpxR. Moreover, our results confirmed the molecular modelling predictions, thereby demonstrating that the presence of D31 in the active site pocket of YeLpxR causes steric hindrance for the binding and deacylation of lipid A species modified with aminoarabinose.

### Regulation of *lpxR* expression

In YeO8 the expression of the loci responsible for the lipid A modification with aminoarabinose, *ugd* and *pmrF* operon, is temperature regulated, being higher at 21°C than at 37°C [15]. Mechanistically, this is so because the expression of the positive regulators *phoPQ* and *pmrAB*, which control the expression of *ugd* and the *pmrF* operon, is also higher at 21°C than at 37°C [15]. In turn, the temperature-dependent regulation of *phoPQ* and *pmrAB* is explained by H-NS-dependent negative regulation alleviated by RovA, another major regulator of *Yersinia* [22], [23], at 21°C [15]. Moreover, there is cross-talk between the regulators in such way that PhoPQ and PmrAB regulate positively the expression of *rovA* and the effect of PhoPQ is more important [15].

**Figure 8:**
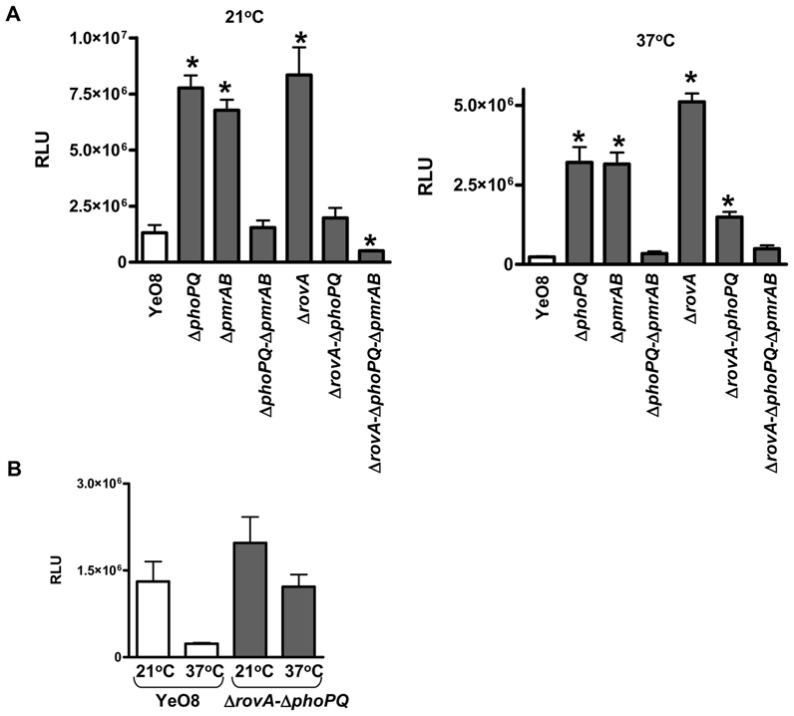
*Y. enterocolitica* PhoPQ, PmrAB two-component systems and RovA control the expression of *lpxR*. (A) Analysis of the expression of *lpxR* by YeO8 (white bar), and mutants (grays bars) YeO8-Δ*phoPQ* (Δ*phoPQ*), YeO8-Δ*pmrAB* (Δ*pmrAB*and YeO8-Δ*phoPQ*-Δ*pmrAB* (Δ*phoPQ-pmrAB*), Yvm927 (Δ*rovA*Yvm927-Δ*phoPQ*-Δ*pmrAB* (Δ*rovA*Δ*phoPQ-*Δ*pmrAB*) carrying the transcriptional fusion *lpxR::lucFF* grown at 21°C or 37°C. Data are presented as mean ± SD (n = 3). *, results are significantly different (p<0.05; two-tailed *t* test) from the results for YeO8 grown at the same temperature. (B) This panel displays the same results shown in panel A for YeO8 and the double mutant Yvm927-Δ*phoPQ* (Δ*rovA*-Δ*phoPQ*) and it is included for the sake of clarity.

The inverse correlation between the substitution of the lipid A with aminoarabinose and lipid A deacylation, prompted us to evaluate whether *phoPQ* and *pmrAB* might negatively regulate *lpxR*. Results shown in figure 8 revealed that the expression of *lpxR::lucFF* was significantly up-regulated in the *phoPQ* and *pmrAB* mutants at 21°C and 37°C (Figure 8A). However, the expression of *lpxR* reached wild-type levels in the double *phoPQ-pmrAB* mutant regardless the bacteria growth temperature (Figure 8A). RT-qPCR experiments showed that the levels of *lpxR* mRNA were higher in the *phoPQ* and *pmrAB* mutants than in the wild type and double *phoPQ-pmrAB* mutants, which were not significantly different (Figure S2).

Recently, we have shown that *rovA* expression is downregulated in the *phoPQ* and *pmrAB* single mutants, being the lowest in the *phoPQ* mutant, whereas in the *phoPQ-pmrAB* double mutant *rovA* expression is not significantly different to that in the wild type [15]. Therefore, the fact that *lpxR* expression follows the opposite trend in these mutants led us to analyze whether *rovA* negatively regulates the expression of *lpxR*. Indeed, luciferase activity was higher in the *rovA* mutant than in the wild type and the levels were not significantly different that those observed in the *phoPQ* mutant when bacteria were grown either at 21°C or 37°C (Figure 8A). Similar results were obtained when the *lpxR* mRNA levels were analyzed by RT-qPCR (Figure S2). The increased *lpxR* expression observed in *rovA* and *phoPQ* single mutants at 21°C was no longer found in the double mutant *rovA-phoPQ* (Figure 8A and Figure S2). When bacteria were grown at 37°C, *lpxR* expression in the *rovA-phoPQ* mutant was significantly lower than those observed in the *rovA* and *phoPQ* single mutants (p<0.05 for each comparison versus *rovA-phoPQ* mutant) although still higher than that in the wild type (Figure 8A and Figure S2). Of note, the expression of *lpxR* was no longer temperature regulated in the *rovA-phoPQ* mutant (Figure 8B). The fact that the expression of *lpxR::lucFF* in the triple mutant *rovA-phoPQ-pmrAB* at 21°C was less than in the wild-type strain may support the notion that, in the absence of the negative regulator RovA, PmrAB and/or a PmrAB-modulated regulator positively regulates *lpxR*. At 37°C, *lpxR* expression in the triple mutant was not significantly different than those found in the double mutant *phoPQ-pmrAB* and the wild type (Figure 8A and Figure S2).

Collectively, our data revealed that the expression of *lpxR* is negatively controlled by the same regulators that activate the loci necessary for the substitution of the phosphate at the 4′ end of the glucosamine disaccharide with aminoarabinose.

### Flagellar regulon and lipid A acylation

In a previous study, we observed the down regulation of YeO8 virulence factors in mutants lacking the lipid A late acyltransferases LpxM, LpxL or LpxP [14]. These results raised the possibility that lipid A acylation may act as a regulatory signal by acting on a transduction pathway(s) [14]. In this context, we sought to determine the impact of LpxR to the expression/function of YeO8 virulence factors.

**Figure 9:**
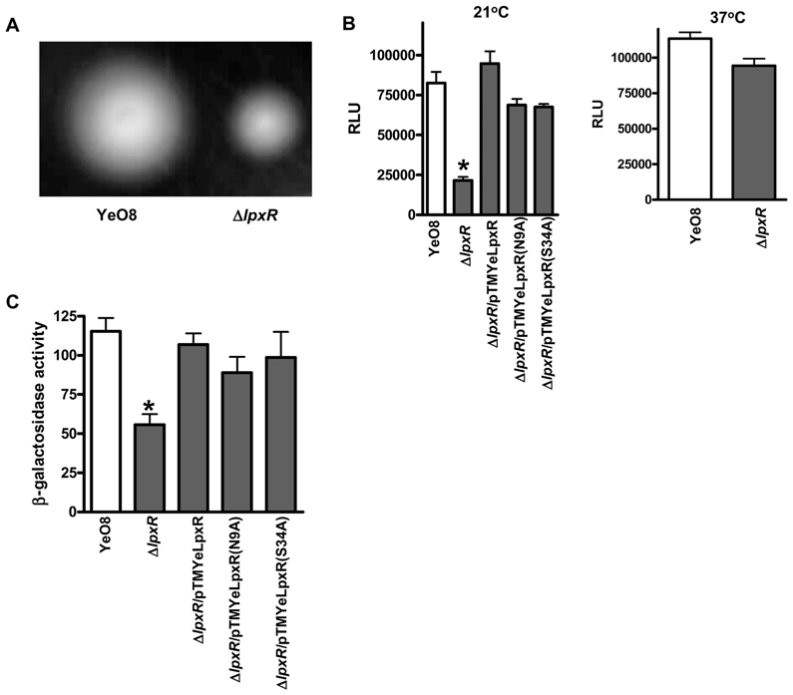
Flagellar regulon is downregulated in the *Y. enterocolitica* O:8 *lpxR* mutant. (A) Motility assays were performed with YeO8, and YeO8-Δ*lpxR*Km (Δ*lpxR*) in a semisolid agar plate (3% agar and 1% tryptone). Plates were incubated at 22°C for 24 h. (B) Analysis of *flhDC* expression by YeO8, YeO8-Δ*lpxR*Km (Δ*lpxR*), and YeO8-Δ*lpxR*Km with the plasmids pTMYeLpxR (Δ*lpxR/*pTMYeLpxR); pTMYeLpxR(N9A) (Δ*lpxR/*pTMYeLpxR(N9A), and pTMYeLpxR(S34A) (Δ*lpxR/*pTMYeLpxR(S34A) carrying the transcriptional fusion *flhDC::lucFF* grown at 21°C and 37°C. (C) β-galactosidase activity production by *yplA'::lacZYA* present in YeO8, YeO8-Δ*lpxR*Km (Δ*lpxR*), and YeO8-Δ*lpxR*Km with the plasmids pTMYeLpxR (Δ*lpxR/*pTMYeLpxR); pTMYeLpxR(N9A) (Δ*lpxR/*pTMYeLpxR(N9A), and pTMYeLpxR(S34A) (Δ*lpxR/*pTMYeLpxR(S34A) [β-galactosidase values given in Miller units, mean ± SD (n = 3)]. *, results are significantly different (p<0.05; two-tailed *t* test) from the results for YeO8.

Virulence genes can be regulated as part of the flagellar regulon, indicating that this regulon contributes to *Y. enterocolitica* pathogenesis [24]. YeO8 is motile when grown at 21°C but not at 37°C [25] and previously we showed that LpxM and LpxP mutants are less motile than the wild type [14]. We examined the influence of LpxR on the flagellar regulon. We quantified the migration of the wild type and YeO8-Δ*lpxR*Km in motility medium (1% tryptone-0.3% agar plates). Figure 9 shows that YeO8-Δ*lpxR*Km was less motile than the wild type. *Yersinia* motility is related to the levels of flagellins which, in turn, are regulated by the expression of *flhDC*, the flagellum master regulatory operon [25], [26]. We hypothesized that the expression of *flhDC* could be lower in the *lpxR* mutant than in the wild type. To address this, the *flhDC::lucFF* transcriptional fusion [26] was introduced into the chromosome of the strains and the luciferase activity was determined. At 21°C, luminescence was lower in the *lpxR* mutant than in the wild type (Figure 9B). Complementation of the *lpxR* mutant with pTMYeLpxR restored *flhDC::lucFF* expression to wild-type levels (Figure 9B). Notably, the catalytic inactive LpxR mutants LpxR(N9A) and LpxR(S34A), encoded by pTMLpxR(N9A) and pTMLpxR(S34A) respectively, also complemented the *lpxR* mutant (Figure 9B). Western blot analysis of purified membranes from YeO8-Δ*lpxR*Km containing pTMLpxR(N9A)FLAG or pTMLpxR(S34A)FLAG showed that the mutant proteins were expressed (Figure S3).When the strains were grown at 37°C, YeO8 and YeO8-Δ*lpxR*Km produced the same luminescence (Figure 9B).

One virulence gene that is regulated as part of the flagellar regulon is *yplA* and hence its expression is regulated by *flhDC* [24], [27], [28]. Considering that *flhDC* expression was downregulated in the *lpxR* mutant, we speculated that *yplA* expression could be affected in this mutant. The transcriptional fusion *yplA::lacZYA* [29] was introduced into the chromosome of the wild type and the *lpxR* mutant and their β-galactosidase activities were measured. Indeed, the β-galactosidase activity was lower in YeO8-Δl*pxR*Km than in the wild type (Figure 9C). Plasmids pTMYeLpxR, pTMLpxR(N9A) and pTMLpxR(S34A) complemented the phenotype (Figure 9C).

In summary, these results indicate that the flagellar regulon is downregulated in the *lpxR* mutant with a concomitant decrease in motility and downregulation of *yplA* expression.

### Invasin and lipid A acylation

**Figure 10:**
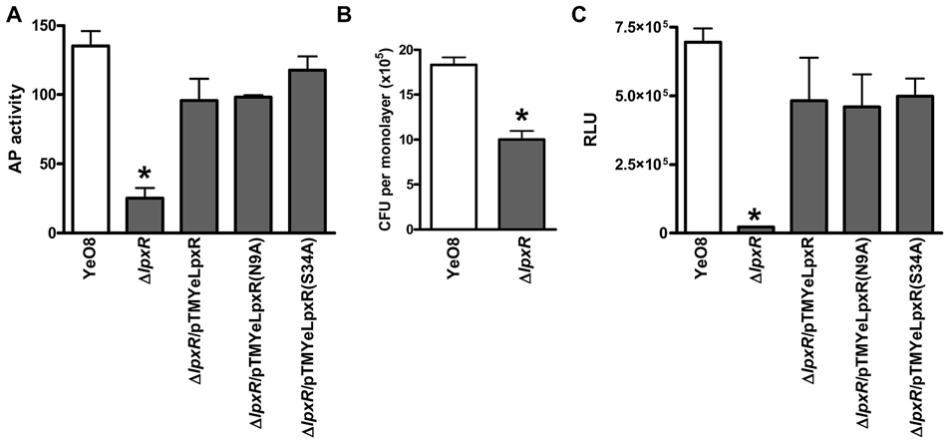
*Inv* expression is altered in *Y. enterocolitica* O:8 *lpxR* mutant. (A) Alkaline phosphatase (AP) activities exhibited by *inv::phoA* translational fusion present in YeO8, YeO8-Δ*lpxR*Km (Δ*lpxR*), and YeO8-Δ*lpxR* with the plasmids pTMYeLpxR (Δ*lpxR/*pTMYeLpxR); pTMYeLpxR(N9A) (Δ*lpxR/*pTMYeLpxR(N9A), and pTMYeLpxR(S34A) (Δ*lpxR/*pTMYeLpxR(S34A) [AP is expressed in enzyme units per OD_600_ unit; mean ± SD (n = 3)]. (B) Invasion of HeLa cells by YeO8, and YeO8-Δ*lpxR*Km (Δ*lpxR*). Invasion assays were done in triplicate without centrifugation (n = 3). (C) Analysis of *rovA* expression by YeO8, YeO8-Δ*lpxR*Km (Δ*lpxR*), and YeO8-Δ*lpxR* with the plasmids pTMYeLpxR (Δ*lpxR/*pTMYeLpxR); pTMYeLpxR(N9A) (Δ*lpxR/*pTMYeLpxR(N9A), and pTMYeLpxR(S34A) (Δ*lpxR/*pTMYeLpxR(S34A) carrying the transcriptional fusion *rovA::lucFF*. *, results are significantly different (p<0.05; one-tailed *t* test) from the results for YeO8.

Inv is an outer membrane protein of *Y. enterocolitica* responsible for invasion of the host [30], [31]. Since YeO8 lipid A mutations affect *inv* expression [14], we asked whether *inv* expression is altered in the *lpxR* mutant. An *inv::phoA* translational fusion [32] was introduced into the genome of YeO8 and YeO8-Δ*lpxR*Km and *inv* expression was monitored as alkaline phosphatase (AP) activity (Figure 10A). AP activity was significantly lower in the *lpxR* mutant than in the wild type. Plasmids pTMYeLpxR, pTMLpxR(N9A) and pTMLpxR(S34A) restored AP activity to wild-type levels. These differences in *inv* expression prompted us to study the ability of YeO8-Δ*lpxR*Km to invade HeLa cells by using a gentamicin protection assay. The amount of intracellular bacteria was 55% lower when cells were infected with the *lpxR* mutant than with the wild type (Figure 10B).

RovA is required for *inv* expression in *Y. enterocolitica* [33]. Therefore, among other possibilities, the low *inv* expression found in the *lpxR* mutant could be caused by downregulation of *rovA* expression. To address this, the *rovA::lucFF* transcriptional fusion [14] was introduced into the genome of the wild type and the *lpxR* mutant and the luminescence was determined. Results shown in figure 10C demonstrate that *rovA* expression was dowregulated in YeO8-Δl*pxR*Km. This phenotype was complemented with plasmids pTMYeLpxR, pTMLpxR(N9A) and pTMLpxR(S34A).

Together, our data show that the down-regulation of *inv* expression found in the *lpxR* mutant is most likely caused by downregulation of *rovA* expression, the positive transcriptional regulator of *inv*.

### Impact of lipid A acylation on pYV-encoded virulence factors

**Figure 11:**
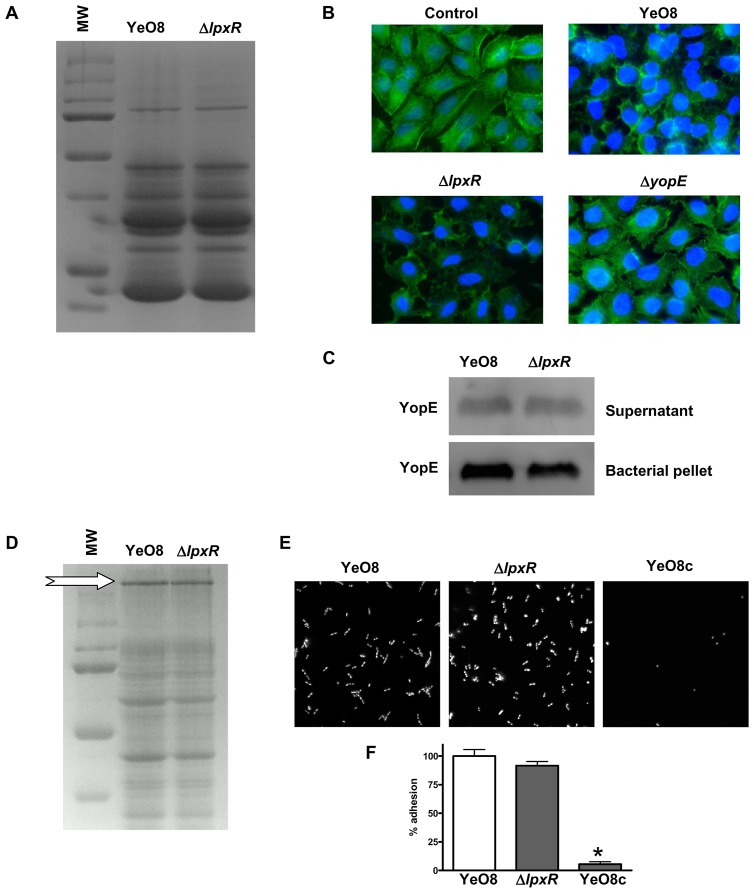
The productions of Yops and YadA are not affected in *Y. enterocolitica* O:8 *lpxR* mutant. (A) SDS-PAGE (the acrylamide concentration was 4% in the stacking gel and 12% in the separation one) and Coomasie brilliant blue staining of proteins from the supernatants of Ca^2+^- deprived cultures from YeO8 and YeO8-Δ*lpxR*Km. Result is representative of four independent experiments. (B) Actin disruption by *Yersinia* infection. A549 cells (monolayer of 70% confluence) were infected with YeO8, YeO8-Δ*lpxR*Km or YeO8-Δ*yopE* (MOI 25∶1) for 1 h. After fixing and permeabilization of cells actin was stained with OregonGreen 514-phalloidin (1∶100) and cells were analyzed by fluorescence microscopy. Result is representative of four independent experiments. (C) Translocation of YopE into A549 cells by YeO8, or YeO8-Δ*lpxR*Km (Δ*lpxR*) (MOI 25∶1 and 1 h of infection). After digitonin extraction, aliquots corresponding to approximately 6×10^4^ infected A549 cells were analysed by SDS-polyacrylamide gel electrophoresis and Western blotting using rabbit polyclonal antiserum raised against YopE (1∶2000 dilution). Result is representative of four independent experiments. (D) SDS-PAGE (the acrylamide concentration was 4% in the stacking gel and 10% in the separation one) followed by Coomasie brilliant blue staining of cell extracts from strains grown in RPMI 1640 at 37°C. White arrow marks YadA protein. Result is representative of four independent experiments. (E) *Y. enterocolitica* strains were allowed to adhere to collagen-coated coverslips. Weakly-bound bacteria were washed off and adherent bacteria stained with Hoechst 33342. YeO8c, pYV-cured derivative of YeO8 (Table 1). (F) Adhering bacteria to collagen-coated coverslips were counted. Wild-type bacteria (YeO8) adherence was set to 100%. Bars represent mean ± SD (n = 4). *, results are significantly different (p<0.05; two-tailed *t* test) from the results for YeO8.

*Y. enterocolitica* harbours a plasmid (pYV)-encoded type III secretion system which is required for virulence. A set of virulence factors, called Yops, are secreted by this system and enable *Y. enterocolitica* to multiply extracellularly in lymphoid tissues [34]–[36]. In several pathogens, LPS polysaccharide status affects the expression of the type III secretion systems [37]–[39]. Therefore, we asked whether the production of the *Yersinia* pYV-encoded type III secretion system is altered in the *lpxR* mutant. At 37°C and under low calcium concentrations, this system secretes the Yops to the culture supernatant [40]. Analysis of Yop secretion revealed that the wild type and the *lpxR* mutant secreted similar levels of Yops (Figure 11A). We sought to determine whether the translocation of Yops to the cytosol of eukaryotic cells is affected in the *lpxR* mutant. Detection of cytoskeleton disturbances upon infection of epithelial cells is one of the most sensitive assays to establish Yop translocation [41]. The injection of YopE into the cytosol of A549 cells by wild-type bacteria induced disruption and condensation of the actin microfilament structure of the cells whereas this was not the case when cells were infected with YeO8-Δ*yopE* mutant (Figure 11B). YopE translocation to A549 cells was not affected in the *lpxR* mutant background (Figure 11C). As expected, A549 cells infected with YeO8-Δl*pxR*Km displayed similar cytoskeleton disturbances than those cells infected with the wild type (Figure 11B).

*yadA* is another pYV-encoded virulence gene whose expression is only induced at 37°C [42]. YadA is an outer membrane protein mediating bacterial adhesion, bacterial binding to proteins of the extracellular matrix and complement resistance (for a review see [43]). Analysis of YadA expression by SDS-PAGE demonstrated that YeO8-Δ*lpxR*Km and YeO8 produced the same amount of the protein (Figure 11D). To assess YadA functionality, we asked whether the YadA-dependent binding to collagen is altered in the *lpxR* mutant. To this end, we analyzed the binding of YadA-expressing whole bacteria to collagen type I by immunofluorescence (see Material and Methods). In contrast to the negative control, a pYV-cured strain (YeO8c), YeO8 and YeO8-Δl*pxR*Km bound to collagen without differences between them (Figure 11E–F).

Taken together, these results suggest that the production and function of the pYV-encoded virulence factors Yops and YadA are not altered in the *lpxR* mutant.

### Lipid A acylation and innate immunity

**Figure 12:**
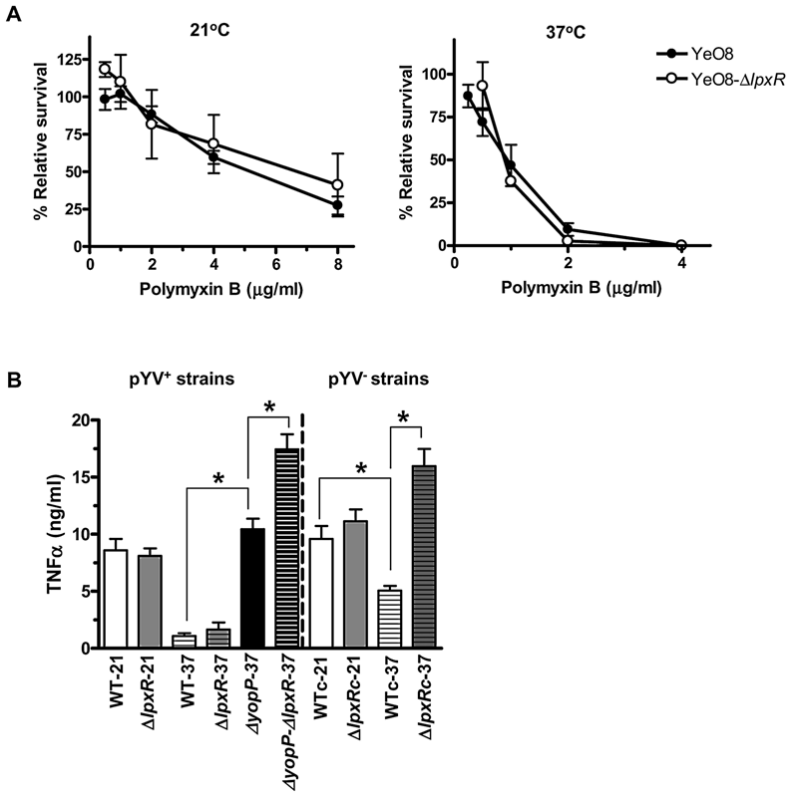
Impact of *lpxR* on *Y. enterocolitica* O:8 interplay with the innate immune system. (A) YeO8 (black circle) or YeO8-Δ*lpxR*Km (white circle) grown at 21°C or 37°C were exposed to different concentrations of polymyxin B. Each point represents the mean and standard deviation of eight samples from four independently grown batches of bacteria. (B) TNFα secretion by infected macrophages with YeO8 (WT), YeO8-Δ*lpxR*Km (Δ*lpxR*), YeO8-Δ*yoP* (Δ*yopP*), YeO8- Δ*yopP*-Δ*lpxR*Km (Δ*yopP*-Δ*lpxR*).“c” denotes bacteria without the virulence plasmid. Strains were grown at 21°C (denoted as 21) and 37°C (denoted as 37). The data are means and s.e.m. *, p<0.05 (for the indicated comparisons).

Cationic antimicrobial peptides (CAMPs) belong to the arsenal of weapons of the innate immune system against infections. In the case of Gram-negative bacteria, CAMPs interact with the lipid A moiety of the LPS [44]–[47] and lipid A modification is one of the strategies employed by Gram-negative bacteria to counteract the action of CAMPs. We and others have used polymyxin B as a model CAMP since it also binds to lipid A. Furthermore, resistance to this peptide reflects well the resistance to other mammalian peptides and correlates with virulence [48]–[51]. Therefore we evaluated the resistance of the *lpxR* mutant to polymyxin B. Results shown in figure 12A demonstrate that the mutant was as resistant as the wild type to the peptide when grown either at 21°C or at 37°C. Of note both strains were more susceptible to polymyxin B when grown at 37°C than at 21°C (Figure 12A).

The mammalian immune system recognizes and responds to *E. coli* LPS via the TLR4 complex, resulting in the synthesis and secretion of pro-inflammatory cytokines that recruit immune cells to the site of infection. The ability of LPSs to evoke inflammatory responses and the potency of them are directly related to the structure of the molecule. It has been reported that underacylated LPSs are less inflammatory than hexa-acylated ones, being the *E coli* lipid A (*m/z* 1797) the prototype of hexa-acylated LPSs [52]. Therefore, the dramatic changes in lipid A acylation displayed by the *lpxR* mutant at 37°C led us to evaluate the immunostimulatory properties of YeO8 and YeO8-Δ*lpxR*Km. As cellular read-out, we determined TNFα levels secreted by macrophages infected either with the wild type or the *lpxR* mutant grown at 21°C and 37°C. YeO8 and YeO8-Δ*lpxR* induced similar levels of TNFα although the levels induced by bacteria grown at 37°C were significantly lower than those triggered by bacteria grown at 21°C (p<0.05 for comparison of TNFα levels between temperatures for a given strain) (Figure 12B). This was dependent on the well known anti-inflammatory action of the pYV-encoded YopP [53], [54], since a *yopP* mutant grown at 37°C induced similar levels of TNFα than those induced by wild-type bacteria grown at 21°C (Figure 12B). Therefore we sought to determine whether YopP could be counteracting the inflammatory response induced by YeO8-Δl*pxR*Km. Indeed, YeO8-Δ*yopP*-Δ*lpxR*Km induced the highest levels of TNFα(Figure 12B). Further sustaining this notion, the TNFα levels induced by the *lpxR* mutant cured of the pYV virulence plasmid grown at 37°C were significantly higher than those induced by the virulence plasmid negative wild-type strain but not different than the YeO8-Δ*yopP*-Δ*lpxR*Km-triggered TNFα levels (Figure 12B). Of note, the TNFα levels induced by the virulence plasmid negative wild-type strain grown at 37°C were significantly lower than those triggered by bacteria grown at 21°C hence further highlighting the importance of lipid A acylation on the immunostimulatory properties of YeO8.

## Discussion

Pathogenic yersiniae show a temperature-dependent variation in lipid A acylation [9]–[14]. At 21°C, *Y. enterocolitica* synthesizes hexa-acylated lipid A containing four 3-OH-C_14_, one C_12_ and either one C_16∶1_ or one C_14_. At 37°C, *Y. enterocolitica* lipid A presents a tetra-acylated species (*m/z* 1388) and a hexa-acylated one containing four 3-OH-C_14_, one C_12_ and C_14_. In a previous work, we identified and characterized the acyltransfreases, *lpxM*, *lpxL* and *lpxP*, responsible for the addition of C_12_, C_14_ and C_16∶1_, respectively, to lipid A [14]. Moreover, we demonstrated that the expressions of these enzymes are temperature regulated [14]. However, the unique tetra-acyl lipid A found in the wild type grown at 37°C (*m/z* 1388) remained to be explained at the molecular level. We and others have established that this species is consistent with 3′-O-deacylation of lipid A [12], [14], [17]. In this work by combining biochemistry, genetics and molecular modelling we present evidence that LpxR is the lipid A 3′-O-deacylase of *Y. enterocolitica*.

YeLpxR is one of the closest homologues to StLpxR. Despite the presence of StLpxR in the *Salmonella* outer membrane, the bacterium does not produce 3′-O-deacylated lipid A species under any growth conditions tested to date [18]. This has been termed as enzyme latency and similar findings have been reported for the *Salmonella* lipid A 3-*O*-deacylase PagL and *E. coli* PagP [55], [56]. Our data revealed that YeLpxR is also latent in the membrane of YeO8 grown at 21°C. However, this is not a general feature of lipid A deacylases since *H. pylori* LpxR is constitutively active [19]. Several explanations could underlie YeLpxR latency at 21°C. Firstly, we explored whether low temperature may affect the function of the enzyme. The fact that YeLpxR did deacylate *E. coli* lipid A when grown at 21°C does not support that low temperatures grossly inhibit the enzyme activity. Nevertheless, we do not by any means completely rule out that temperature may affect YeLpxR activity, and thorough biochemical analyses are warranted to rigorously define the functional parameters of YeLpxR activity. This will be the subject of future studies. We next hypothesized that specific features of YeO8 lipid A, which do not exist in the *E. coli* lipid A, may be responsible for YeLpxR latency. The first conspicuous difference is the type of secondary fatty attached to the lipid IV_A_. In *E. coli* the late acyltransferases LpxL and LpxM add laureate (C_12_) and myristate (C_14_) respectively [1] whereas in YeO8 these enzymes transfer myristate (C_14_) and laureate (C_12_) respectively [14]. However, this cannot account for the reduced LpxR activity since the enzyme did deacylate *E. coli* lipid A. The presence of palmitoleate in YeO8 lipid A at 21°C but not at 37°C cannot be the reason since YeLpxR deacylated *E. coli* lipid A containing palmitoleate, found in *E. coli* grown at 21°C. Instead, our results revealed that the lipid A substitution with aminoarabinose is associated with YeLpxR latency since LpxR-dependent lipid A deacylation was clearly observed in the *pmrF* mutant grown at 21°C. Notably, the lack of aminoarabinose also releases *Salmonella* PagL from latency [56], hence suggesting a key role for the lipid A modification with aminoarabinose in LPS remodelling.

The molecular modelling and docking experiments further highlighted the importance of lipid A substitution with aminoarabinose for YeLpxR function. D31 in YeLpxR forces the conserved K67 to adopt a different conformation compared to StLpxR. According to the docking results, the resulting loss of cavity space in the vicinity of K67 in YeLpxR, causes the phosphate at the 4′ end of Kdo_2_-lipidA to bind somewhat differently to YeLpxR than to StLpxR. In the latter, the phosphate binds in the cavity near K67, while in YeLpxR it is forced to bind more outwards from the enzyme. The docking of Kdo_2_-lipidA with aminoarabinose to StLpxR showed that aminoarabinose occupies the cavity space, which corresponds to a narrow connection between two larger cavities in YeLpxR. The large reduction in cavity volume at this particular site causes this space to be too small for the accommodation of aminoarabinose. Hence, D31 seems to cause steric hindrance for the binding of aminoarabinose-containing Kdo_2_-lipidA to YeLpxR. Therefore, we predicted that D31 could have an important role for the YeLpxR substrate specificity. Indeed, the site-directed mutagenesis experiments validated that the presence of D31 in the active site pocket of YeLpxR causes a steric hindrance for the binding and deacylation of lipid A species modified with aminoarabinose. Nevertheless, at present we do not rule out that other residues of YeLpxR also contribute to its latency. In this regard, *Salmonella* PagL is released from latency when specific amino acid residues located at extracellular loops of the enzyme are mutated and it has been postulated that these residues are involved in the recognition of aminoarabinose-modified lipid A [56]–[58]. Studies are going to explore whether residues located at extracellular loops of LpxR also contribute to enzyme latency.

The inverse correlation between the aminoarabinose content in the LPS and the LpxR-dependent lipid A deacylation prompted us to evaluate whether the same regulatory network governing the expression of the *pmrF* operon and *ugd* could regulate *lpxR*. Recently, we have shown that the global regulators RovA, PhoPQ, and PmrAB positively control the expression of the loci necessary for aminoarabinose biosynthesis at 21°C [15]. Furthermore, there is a cross-talk between these regulators since the expressions of *phoPQ* and *pmrAB* are downregulated in the *rovA* mutant whereas *rovA* expression is downregulated in *phoPQ* and *pmrAB* single mutants [15]. Our findings support the notion that RovA and PhoPQ are negative regulators of *lpxR* since its expression was higher in *phoPQ* and *rovA* single mutant backgrounds than in the wild type. In turn, the two-component system PmrAB and/or a PmrAB-regulated system may act as a positive regulator because *lpxR* expression was similar in the wild-type and *rovA-phoPQ* backgrounds.

One striking finding of our study is that motility and invasion of eukaryotic cells were reduced in the *lpxR* mutant grown at 21°C. Mechanistically, our data revealed that the expressions of *flhDC* and *rovA*, the key regulators controlling the flagellar regulon and invasin respectively [22], [25], [33], were down-regulated in the *lpxR* mutant. Although we have reported that lipid A acylation status affects motility and invasion [14], the phenotypes were found in mutants lacking the late-acyltransferases and hence displaying major changes in the lipid A structure at 21°C [14]. This is in contrast to the *lpxR* mutant grown at 21°C, where the LpxR-dependent deacylation was hardly observed. The fact that YeLpxR is in latent stage at this growth temperature may suggest that, in the *lpxR* mutant background, the absence of the enzyme in the outer membrane, not the lipid A deacylation, acts as the regulatory signal underlying the reduced expressions of *flhDC* and *rovA*. Given experimental support to this hypothesis, the catalytically inactive mutants LpxR(N9A) and LpxR(S34A) restored the expressions of *flhDC*, *ylpA*, *inv* and *rovA* to wild-type levels. These results are in good agreement with the notion that membrane-intrinsinc β-barrel proteins, such as LpxR, may launch transmembrane signal transduction pathways upon sensing outer membrane perturbations [59], in our case, the absence of the protein itself. Therefore, it can be speculated that those systems sensing extracytoplasmatic stresses could underlie the regulatory connection between the absence of LpxR and the expression of *Y. enterocolitica* virulence factors. Giving indirect support to our speculation, it has been reported that lipid A deacylation induces σ^E^-dependent responses in *E. coli* [60], the Cpx system senses changes in LPS O-polysaccharide [61]. Experiments are underway to test whether the activation status of the Cpx and/or σ^E^ systems is altered in the *lpxR* mutant background and whether any of these systems is responsible for the reduced expression of *flhDC* and *rovA* found in the mutant.

The LPS contains a molecular pattern recognized by the innate immune system thereby arousing several host defence responses. On one hand, CAMPs target this LPS pattern to bind to the bacterial surface, which is necessary for their microbicidal action. On the other hand, recognition of the LPS by the LPS receptor complex triggers the activation of host defence responses, chiefly the production of inflammatory markers. Not surprisingly, the modification of the LPS pattern is a virulence strategy of several pathogens to evade the innate immune system, and *Y. enterocolitica* is not an exception. Recently, we have demonstrated that the temperature-dependent lipid A modifications with aminoarabinose and palmitate help *Y. enterocolitica* to avoid the bactericidal action of CAMPs [15]. In this context, it was not totally unexpected to find out that the *lpxR* mutant was as susceptible as the wild type to polymyxin B, a model CAMP, since the mass spectrometry analysis indicated that the aforementioned lipid A modifications were not affected in the *lpxR* mutant background. Concerning the activation of inflammatory responses, several studies highlight the critical role of pYV-encoded Yops, chiefly YopP, to prevent the activation of inflammatory responses in a variety of cells, including macrophages. Nevertheless, Rebeil and co-workers [12] conclusively demonstrated that purified LPS from *Y. enterocolitca* grown at 37°C is less inflammatory than that purified from bacteria grown at 21°C. This is in agreement with the concept that underacylated LPSs are less inflammatory than hexa-acylated ones [52]. Therefore, it was plausible to speculate that the LpxR-dependent deacylation of LPS at 37°C was responsible for the reduced stimulatory potential of the LPS described by Rebeil and co-workers. To confirm this speculation we chose to challenge macrophages with alive bacteria instead of using purified LPS since there might be differences between the cellular recognition of purified LPS and the LPS expressed in the complex lipid environment of the bacterial outer membrane. To our initial surprise, we observed that the *lpxR* mutant elicited similar inflammatory response than the wild type when both strains were grown at 37°C. The fact that these responses were significantly lower than those elicited by bacteria grown at 21°C suggested that pYV-encoded factors were attenuating the inflammatory response. Therefore, we hypothesized that the arsenal of Yops injected to the cell were efficiently counteracting the activation of inflammatory responses evoked by the *lpxR* mutant LPS. In fact, our data demonstrated that the production and function of the pYV-encoded virulence factors were not affected in the *lpxR* mutant. Giving support to our hypothesis, the inflammatory response elicited by the *lpxR* mutant cured of the pYV virulence plasmid grown at 37°C was significantly higher than that induced by the virulence plasmid negative wild-type strain. Moreover, our findings suggest that, among all Yops, YopP plays a major role in counteracting the inflammation elicited by the *lpxR* mutant since the TNFα levels induced by the *lpxR* mutant cured of the pYV virulence plasmid grown at 37°C were not different than those triggered by YeO8-Δ*yopP*-Δ*lpxR*. On the whole, our results and those reported by Rebeil and co-workers [12] are consistent with a model in which the characteristic low inflammatory response associated to *Y. enterocolitica* infections might be the sum of the anti-inflammatory action exerted by YopP and the reduced activation of the LPS receptor complex due to the expression of a LpxR-dependent deacylated LPS. In this scenario, the latency of LpxR may facilitate a quick bacterial response upon entering the host to reduce the initial recognition of the pathogen by the LPS receptor complex. This will allow the pathogen to activate other host countermeasures, among others the pYV-encoded type III secretion system, which is a time consuming process.

## Materials and Methods

### Bacterial strains and growth conditions

Bacterial strains and plasmids used in this study are listed in Table 1. Unless otherwise indicated, *Yersinia* strains were grown in lysogeny broth (LB) medium at either 21°C or 37°C. When appropriate, antibiotics were added to the growth medium at the following concentrations: ampicillin (Amp), 100 µg/ml for *Y. enterocolitica* and 50 µg/ml for *E. coli*; kanamycin (Km), 100 µg/ml in agar plates for *Y. enterocolitica*, 50 µg/ml in agar plates for *E. coli*, and 20 µg/ml in broth; chloramphenicol (Cm), 20 µg/ml; trimethoprim (Tp), 100 µg/ml; tetracycline (Tet) 12.5 µg/ml; and streptomycin (Str), 100 µg/ml.

### *Y. enterocolitica* mutant construction

*In silico* analysis led to the identification of *Y. enterocolitica* 8081 homologue of *lpxR* (YE3039), *yopP* (YEP0083) and *yopE* (YEP0053) [accession number AM286415; [20]]. To obtain the *lpxR*, *yopP*, and *yopE* mutants two sets of primers (Table S1) were used for each gene to amplify two different fragments from each gene, LpxRUP and LpxRDOWN, YopPUP and YopPDOWN, YopEUP and YopEDOWN, respectively. Both fragments were BamHI-digested, purified, ligated, amplified as a single PCR fragment using a mixture of Go*Taq Flexi* polymerase (2.5 units/reaction; Promega) and *Vent* polymerse (2.5 units/reaction; New England Biolabs), gel purified and cloned into pGEMT-Easy (Promega) to obtain pGEMTΔ*lpxR*, pGEMTΔ*yopP*, and pGEMTΔ*yopE* respectively. A kanamycin resistance cassette flanked by FRT recombination sites was obtained as a BamHI fragment from pGEMTFRTKm and it was cloned into BamHI-digested pGEMTΔ*lpxR* and pGEMTΔ*yopP* to generate pGEMTΔ*lpxR*Km and pGEMTΔ*yopP*Km respectively. Δ*lpxR*::Km, and Δ*yopP*::Km alleles were amplified using *Vent* polymerase (New England Biolabs) and cloned into SmaI-digested pKNG101 to obtain pKNGΔ*lpxR*Km and pKNGΔ*yopP*Km, respectively. Δ*yopE* allele was obtained by PvuII-digestion of pGEMTΔ*yopE*, gel purified and cloned into SmaI-digested pKNG101 to obtain pKNGΔ*yopE*. pKNG101 is a suicide vector that carries the defective *pir-*negative origin of replication of R6K, the RK2 origin of transfer, and an Str resistance marker [62]. It also carries the *sacBR* genes that mediate sucrose sensitivity as a positive selection marker for the excision of the vector after double crossover [62]. Plasmids were introduced into *E. coli* CC118-λ*pir* from which they were mobilized into *Y. enterocolitica* 8081 by triparental conjugation using the helper strain *E. coli* HB101/pRK2013. Bacteria were diluted and aliquots spread on *Yersinia* selective agar medium plates (Oxoid) supplemented with Str. Bacteria from 5 individual colonies were pooled and allowed to grow in LB without any antibiotic overnight at RT. Bacterial cultures were serially diluted and aliquots spread in LB without NaCl containing 10% sucrose and plates were incubated at RT. The recombinants that survived 10% sucrose were checked for their antibiotic resistance. The appropriate replacement of the wild-type alleles by the mutant ones was confirmed by PCR and Southern blot (data not shown). In the case of YeO8-Δ*lpxR*Km and YeO8-Δ*yopP*Km mutants, the kanamycin cassette was excised by Flp-mediated recombination [63] using plasmid pFLP2Tp. This plasmid is a derivative from pFLP2 constructed by cloning a trimethoprim resistance cassette, obtained by SmaI digestion of p34S-Tp [64], into ScaI-digested pFLP2. The generated mutants were named YeO8-Δ*lpxR* and YeO8-Δ*yopP*, respectively. YeO8-Δ*yopP*-Δ*lpxR*Km and YeO8-Δ*pmrF*-Δ*lpxR*Km double mutants were obtained mobilizing the pKNGΔ*lpxR*Km plasmid into YeO8-Δ*yopP* and YeO8-Δ*pmrF*, respectively. The replacement of the wild-type alleles by the mutant ones was done as described above and confirmed by PCR (data not shown).

To cure the pYV plasmid from YeO8-Δ*lpxR*Km, bacteria were grown at 37°C in Congo Red Magnesium oxalate agar plates [65]. Colony size and lack of uptake of Congo Red were used to detect loss of the virulence plasmid. This was further confirmed by testing the YadA-dependent autoagglutination ability [66].

### Construction of *lpxR::lucFF* reporter fusion

A 443 bp DNA fragment containing the promoter region of *lpxR* was amplified by PCR using *Vent* polymerase (see Table S1 for primers used), EcoRI digested, gel purified and cloned into EcoRI-SmaI digested pGPL01Tp suicide vector [15]. This vector contains a promoterless firefly luciferase gene (*lucFF*) and a R6K origin of replication. A plasmid in which *lucFF* was under the control of the *lpxR* promoter was identified by restriction digestion analysis and named pGPL01TpYelpxR. This plasmid was introduced into *E. coli* DH5α-λ*pir* from which it was mobilized into *Y. enterocolitica* by triparental conjugation using the helper strain *E. coli* HB101/pRK2013. Strains in which the suicide vectors were integrated into the genome by homologous recombination were selected. This was confirmed by PCR (data not shown).

### Complementation of *lpxR* mutant

To complement the *lpxR* mutant, a DNA fragment of 1.5 kb was PCR-amplified using TaKaRa polymerase (see Table S1 for primers used) gel purified, and cloned into pGEMT-Easy (Promega) to obtain pGEMTCom*lpxR*. A fragment, containing the putative promoter and coding region of the deacylase, was obtained by PvuII digestion of pGEMTCom*lpxR*, gel purified and cloned into the ScaI site of the medium copy plasmid pTM100 [40] to obtain pTMLpxR. For the construction of plasmid pTMLpxRFLAG, the *lpxR* coding region with its own promoter and a FLAG epitope sequence right before the stop codon was PCR amplified using *Vent* polymerase, primers LpxRtagging and LpxrFLAG (Table S1) and genomic DNA as template. The fragment was phosphorylated, gel purified and cloned into ScaI-digested pTM100. pTMLpxR and pTMLpxRFLAG were introduced into *E. coli* DH5α-λ*pir* and then mobilized into *Y. enterocolitica* strains by triparental conjugation using the helper strain *E. coli* HB101/pRK2013.

### Isolation and analysis of lipidA

Lipid As were extracted using an ammonium hydroxide/isobutyric acid method and subjected to negative ion matrix-assisted laser desorption ionization time-of-flight (MALDI-TOF) mass spectrometry analysis [14], [67]. Analyses were performed on a Bruker Autoflex II MALDI-TOF mass spectrometer (Bruker Daltonics, Incorporated) in negative reflective mode with delayed extraction. Each spectrum was an average of 300 shots. The ion-accelerating voltage was set at 20 kV. Dihydroxybenzoic acid (Sigma Chemical Co., St. Louis, MO) was used as a matrix. Further calibration for lipid A analysis was performed externally using lipid A extracted from *E. coli* strain MG1655 grown in LB at 37°C. Interpretation of the negative-ion spectra is based on earlier studies showing that ions with masses higher than 1000 gave signals proportional to the corresponding lipid A species present in the preparation [9], [12], [17], [68]. Important theoretical masses for the interpretation of peaks found in this study are: lipid IV_A_, 1405; C_12_, 182, C_14_, 210; C_16∶1_, 236.2; aminoarabinose (AraNH), 131.1; C_16_, 239.

### Site-directed mutagenesis

Site-directed mutagenesis of the *lpxR* gene was performed by PCR [69]. Plasmid pTMLpxR, obtained with a minipreparation kit (Macherey-Nagel), was used as template and the desired mutations were introduced by the primer pairs described in Table S1. Amplifications were carried out in 50 µl reaction mixture using *Vent* DNA polymerase (New England BioLabs.). The PCR was started with initial 70 sec incubation at 95°C and then steps (95°C 50 sec, 60°C 75 sec and 72°C 6 min) were repeated 20 times followed by a 10 min extension time at 72°C. The obtained PCR products were gel purified, phosphorylated with T4 polynucleotide kinase, ligated, and digested with DpnI to break down any remaining template plasmid. The ligated PCR-product was transformed into *E. coli* C600. Plasmid DNA was isolated from transformants and the *lpxR* gene was completely sequenced to confirm the generated mutations and to ensure that no other changes were introduced. The name of each mutant construct includes the wild-type residue (single-letter amino acid designation) followed by the codon number and mutant residue (typically alanine).

For the construction of plasmids pTMLpxR(N9A)FLAG and pTMLpxR(S34A)FLAG, the *lpxR* alleles encoded into pTMLpxR(N9A) and pTMLpxR(S34A) were PCR amplified using *Vent* polymerase, and primers LpxRtagging and LpxrFLAG (Table S1). The fragments were phosphorylated, gel purified and cloned into ScaI-digested pTM100 [40]. Plasmids were introduced into *E. coli* DH5α-λ*pir* and then mobilized into *Y. enterocolitica* strains by triparental conjugation using the helper strain *E. coli* HB101/pRK2013.

### Purification of membrane proteins and Western blot analysis of LpxR FLAG tagged levels

Overnight 5-ml cultures of *Y. enterocolitica* strains were diluted 1∶21 into 100 ml of LB in a 250-ml flask. Cultures were incubated with aeration at 21°C or 37°C until OD_600_ 0.8. Bacteria were recovered by centrifugation (6500×*g*; 10 min, RT) and they were resuspended in 2 ml of 10 mM Tris/HCl (pH 7.4)-5 mM MgSO4 containing 2% Triton X-100 (v/v). Cells were broken by sonication (Branson digital sonifier; microtip 1/8″ diameter, amplitude 10%) for 15×1 min cycles, each cycle comprised 1 min sonication step separated by 1 min intervals. Unbroken cells were eliminated by centrifugation (2000×*g*, 20 min), and cell envelopes were recovered by ultracentrifugation (Beckman 70.1 Ti rotor; 45 000×*g*; 1 h, 4°C). The cell envelopes were resuspended in 500 µl of distilled water. The protein concentration was determined using the BCA Protein Assay Kit (Thermo Scientifc).

80 µg of proteins were separated on 4–12% SDS-PAGE, and semi-dry electrotransferred onto a nitrocellulose membrane using as transfer buffer SDS-PAGE-urea lysis buffer [a freshly prepared 1∶1 mix of 1× SDS running buffer (12 mM Tris, 96 mM glycine, 0.1% SDS] and urea lysis buffer (10 mM Na_2_HPO_4_, 1% β-mercaptoethanol, 1%SDS, 6 M urea)] [70]. Membrane was blocked with 4% skim milk in PBS. Membranes were stained using anti-Flag antibody (1∶2000; Sigma) following the instructions of the supplier.

### Structural modeling of *Yersinia enterocolitica* LpxR

A homology model of YeLpxR was constructed based on the crystal structure of StLpxR (PDB code 3FID; [21]. The YeLpxR sequence was used as bait to search Protein Data Bank with the Basic Local Alignment Search Tool (BLAST) at NCBI (http://blast.ncbi.nlm.nih.gov/). A pairwise sequence alignment was made using the program MALIGN [71] in the BODIL modeling environment [72], and a picture of the alignment was created using ESPript [73]. The essential water molecule and the zinc ion in the StLpxR crystal structure were also included in the YeLpxR model. A set of ten models was created with the program MODELLER [74], from which the model with the lowest value of the MODELLER objective function was analyzed and compared to the crystal structure of StLpxR by superimposing with the program VERTAA (Johnson & Lehtonen, 2004) in BODIL. Different rotamers for D10 and D31 were searched with the program Jackal (http://wiki.c2b2.columbia.edu/honiglab_public/index.php/Software:Jackal). D10 was changed to the same rotamer as in the crystal structure of StLpxR, while the rotamer used for D31 was the one with the lowest energy according to Jackal. SURFNET [75] was used to detect surface cavities, while PyMOL (Version 1.4, Schrödinger, LLC) was used for preparing pictures. For the SURFNET calculations, the minimum radius for gap spheres was set to 1.5 Å and the maximum radius was 4.0 Å.

For the docking studies, a Kdo_2_-lipid A, both with and without aminoarabinose, was modified from the coordinates for the LPS molecule in the crystal structure of FhuA [76]. The fatty acyl chains were removed from the Kdo_2_-lipid A molecule in order to reduce the number of rotatable bonds and make the docking more reliable. Aminoarabinose was added to the modified Kdo_2_-lipid A molecule with SYBYL (Version 8.0, Tripos Associates, Inc., St Louis, MO, USA), and the structure was minimized with the conjugate gradient method and Tripos force field. The modified Kdo_2_-lipid A, both with and without aminoarabinose, was docked to the YeLpxR model and the StLpxR crystal structure (PDB code 3FID) with GOLD via Discovery Studio (CSC IT Center for Science Ltd, Espoo, Finland), with default docking parameters and the receptor cavity defined to D10, Q16, T/S34, K67, and Y130.

### Luciferase activity

The reporter strains were grown at 21°C or at 37°C on an orbital incubator shaker (180 r.p.m.) until OD_540_ 1.6. The cultures were harvested (2500×g, 20 min, 24°C) and resuspended to an OD_540_ of 1.0 in PBS. A 100 µl aliquot of the bacterial suspension was mixed with 100 µl of luciferase assay reagent (1 mM D-luciferin [Synchem] in 100 mM citrate buffer pH 5). Luminescence was immediately measured with a Luminometer LB9507 (Berthold) and expressed as relative light units (RLU). All measurements were carried out in quintuplicate on at least three separate occasions.

### Analysis of motility and *flhDC* expression

Phenotypic assays for swimming motility were initiated by stabbing 2 µl of an overnight culture at the centre of agar plates containing 0.3% agar and 1% tryptone [25], [26]. Plates were analyzed after 24 h of incubation at RT and the diameters of the halos migrated by the strain from the inoculation point were compared. Experiments were run in quadruplicate in three independent occasions.

To measure *flhDC* expression, plasmid pRSFlhDC08 [26] encoding the transcriptional fusion *flhDC::lucFF* was integrated into the genomes of the strains by homologous recombination. This was confirmed by Southern blot (data not shown). Luminescence was determined as previously described.

### β-galactosidase and alkaline phosphatase activities

β-galactosidase activity was determined as previously described with bacteria grown in 1% tryptone at RT [77]. Alkaline phosphatase activity was determined in permeabilized cells and the results are expressed in enzyme units per OD_600_ as previously described [78]. Experiments were run in duplicate in three independent occasions.

### Real time-quantitative PCR (RT-qPCR)

Bacteria were grown at 21°C or at 37°C in 5 ml of LB medium on an orbital incubator shaker (180 r.p.m.) until an OD_600_ of 0.3. 0.5 ml of ice-cold solution EtOH/phenol [19∶1 v/v (pH 4.3)] were added to the culture and the mixture was incubated on ice for 30 min to prevent RNA degradation. Total RNA was extracted using a commercial NucleoSpin RNA II kit as recommended by the manufacturer (Macherey-Nagel).

cDNA was obtained by retrotranscription of 2 µg of total RNA using a commercial M-MLV Reverse Transcriptase (Sigma), and random primers mixture (SABiosciences, Quiagen). 50 ng of cDNA were used as a template in a 25-µl reaction. RT-PCR analyses were performed with a Smart Cycler real-time PCR instrument (Cepheid, Sunnyvale, CA) and using a KapaSYBR Fast qPCR Kit as recommended by the manufacturer (Cultek). The thermocycling protocol was as follows; 95°C for 3 min for hot-start polymerase activation, followed by 45 cycles of 95°C for 15 s, and 60°C for 30 s. SYBR green dye fluorescence was measured at 521 nm.

cDNAs were obtained from three independent extractions of mRNA and each one amplified by RT-qPCR in two independent occasions. Relative quantities of *lpxR* mRNAs were obtained using the comparative threshold cycle (*ΔΔ*C_T_) method by normalizing to *rpoB* and *tonB* genes (Table S1).

### Analysis of Yop secretion

Overnight cultures of *Y. enterocolitica* strains were diluted 1∶50 into 25 ml of TSB supplemented with 20 mM MgCl_2_ and 20 mM sodium oxalate in a 100-ml flask. Cultures were incubated with aeration at 21°C for 2.5 h, and then transferred at 37°C for 3 h. The optical density at 540 nm of the culture was measured and the bacterial cells were collected by centrifugation at 1500×*g* for 30 min. Ammonium sulphate (final concentration 47.5% w/v) was used to precipitate proteins from 20 ml of the supernatant. After overnight incubation at 4°C, proteins were collected by centrifugation (3000×*g*, 30 min, 4°C) and washed twice with 1.5 ml of water. Dried protein pellets were resuspended in 50 to 80 µl of sample buffer and normalized according to the cell count. Samples were analyzed by sodium dodecyl sulfate-polyacrylamide gel electrophoresis (SDS-PAGE) using 12% polyacrylamide gels and proteins visualized by Coomassie brilliant blue staining.

Control experiments revealed that the secretion of Yops was not affected in *yopE* and *yopP* mutants except that each mutant did not produce either YopE or YopP, respectively (data not shown).

### Analysis of YadA production

Bacteria were grown overnight in 2 ml RPMI 1640 medium lacking phenol red at 37°C without shaking. The OD_540_ of the culture was measured and CFUs were determined by plating serial dilutions. Bacteria from 1-ml aliquot were recovered by centrifugation (16 000×*g*, 10 min, 4°C) and resuspended in 200 µl of SDS-sample buffer. Samples were incubated for 4 h at 37°C and kept frozen at −20°C. Samples were analyzed by SDS-PAGE using 10% polyacrylamide gels and proteins visualized by Coomassie brilliant blue staining. Samples were normalized according to the cell count and they were not boiled before loading the gel.

### Binding assay to collagen-coated slides

Overnight cultures of *Y. enterocolitica* strains grown at 37°C were diluted 1∶10 into 5 ml of LB and grown with aeration at 37°C for 2.5 h. bacteria were pelleted, washed once with PBS and resuspended to an OD_540_ of 0.3 in PBS.

12 mm circular coverslips in 24-well tissue culture plates were coated overnight at 4°C with 10 µg/ml human collagen type I (Sigma) in PBS (final volume 100 µl). Coverslips were washed three times with TBS and later they were blocked for 1 h at 4°C with 2% BSA in TBS. Finally, coverslips were washed three times and were incubated at 37°C with 100 µl of the bacterial suspension. After 1 h incubation, the coverslips were washed three times with PBS and then bacteria fixed with 3.7% paraformaldehyde (PFA) in PBS pH 7.4 for 20 min at room temperature. PFA fixed cells were incubated with PBS containing 0.1% saponin, 10% horse serum and Hoechst 33342 (1∶25000) for 30 min in a wet dark chamber. Finally, coverslips were washed twice in 0.1% saponin in PBS, once in PBS and once in H_2_O, mounted on Aqua Poly/Mount (Polysciences) and analysed with a Leica CTR6000 fluorescence microscope. Bacteria were counted in images from three randomly selected fields of view obtained at a magnification of ×100 taken with a Leica DFC350FX camera. Wild-type adhesion was set to 100%.

### Actin disruption by *Yersinia* infection

Carcinomic human alveolar basal epithelial cells (A549, ATTC CCL-185) were maintained in RPMI 1640 tissue culture medium supplemented with 1% HEPES, 10% heat inactivated foetal calf serum (FCS) and antibiotics (penicillin and streptomycin) in 25 cm^2^ tissue culture flasks at 37°C in a humidified 5% CO_2_ atmosphere as previously described [79]. For infections, A549 cells were seeded on 12 mm circular coverslips in 24-well tissue culture plates to 70% confluence. Cells were serum starved 16 h before infection. Overnight cultures of *Y. enterocolitica* strains grown at 21°C were diluted 1∶10 into 5 ml of LB and grown with aeration at 21°C for 1.5 h and then 1 h at 37°C. Bacteria were pelleted, washed once with PBS and resuspended to an OD_600_ = 1 (approximately 10^9^ CFU/ml) in PBS. Cells were infected with this suspension to get a multiplicity of infection of 25∶1. After 1 h incubation, the coverslips were washed three times with PBS and then cells fixed with 3.7% PFA in PBS pH 7.4 for 20 min at room temperature. PFA fixed cells were incubated with PBS containing 0.1% saponin, 10% horse serum, Hoechst 33342 (1∶2500), and OregonGreen 514-phalloidin (1∶100) (Invitrogen) for 30 min in a wet dark chamber. Finally, coverslips were washed twice in 0.1% saponin in PBS, once in PBS and once in H_2_O, mounted on Aqua Poly/Mount (Polysciences) and analysed with a Leica CTR6000 fluorescence microscope. Images were taken with a Leica DFC350FX camera.

### YopE translocation

YopE translocation into A549 cells was done as previously described [38]. Briefly, A549 cells were seeded in 12-well tissue culture plates to 80% confluence. Cells were serum starved 16 h before infection. Overnight cultures of *Y. enterocolitica* strains grown at 21°C were diluted 1∶10 into 5 ml of LB and grown with aeration at 21°C for 1.5 h and then 1 h at 37°C. Bacteria were pelleted, washed once with PBS and resuspended to an OD_600_ = 1 (approximately 10^9^ CFU/ml) in PBS. Cells were infected with this suspension to get a multiplicity of infection of 25∶1. To synchronize infection, plates were centrifuged at 200×*g* during 5 min. After 1 h infection, cells were washed twice with PBS and resuspended in 400 µl of PBS with the help of a rubber policeman. Cell suspensions were transferred to a 1.5 ml microcentrifuge tube and cells pelleted (16 000×g; 12 sec). Supernatant was carefully removed and cells were resuspended in 100 µl of 1% digitonin (w/v) in PBS supplemented with a cocktail of protease inhibitors (Halt protease inhibitor single-use cocktail EDTA-free; Thermo). After 2-min incubation at RT, samples were centrifuged (16 000×*g*; 10 min, 4°C). 80 µl of the supernatant, containing cytosolic proteins, were collected to whom 20 µl of 5× SDS sample buffer were added. The pellet, containing intact bacteria and cell membranes, was resuspended in 100 µl 1× SDS sample buffer. Aliquots corresponding to approximately 6×10^4^ infected A549 cells were analysed by SDS-polyacrylamide gel electrophoresis and Western blotting using rabbit polyclonal antiserum raised against YopE (1∶2000 dilution).

### Invasion to HeLa cells

Strains were grown aerobically for 16 h at RT, pelleted and resuspended to an OD_540_ of 0.3 in PBS. Bacteria suspensions were added to subconfluent HeLa cells at a multiplicity of infection of ∼25∶1. After a 30 min infection, monolayers were washed twice with PBS and then incubated for an additional 90 min in medium containing gentamicin (100 µg/ml) to kill extracellular bacteria. This treatment was long enough to kill all extracellular bacteria. After this period, cells were washed three times with PBS and lysed with 0.5% saponin in PBS and bacteria were plated. Experiments were carried out in triplicate on three independent occasions. Invasion is expressed as CFUs per monolayer.

### Polymyxin B susceptibility assay

Bacteria were grown either at 21°C or 37°C in 5 ml LB in a 15-ml Falcon tube with shaking (180 rpm), and harvested (2500×g, 20 min, 24°C) in the exponential growth phase (OD_540_ 0.8). Bacteria were washed once with PBS and a suspension containing approximately 1×10^5^ CFU/ml was prepared in 10 mM PBS (pH 6.5), 1% Tryptone Soya Broth (TSB; Oxoid), and 100 mM NaCl. Aliquots (5 µl) of this suspension were mixed in 1.5 ml microcentrifuge tubes with various concentrations of polymyxin B (Sigma). In all cases the final volume was 30 µl. After 1 h incubation at the bacterial growth temperature, the contents of the tubes were plated on LB agar. Colony counts were determined and results were expressed as percentages of the colony count of bacteria not exposed to antibacterial agents. All experiments were done with duplicate samples on at least four independent occasions.

### Macrophage culture and TNFα ELISA

Murine macrophages RAW264.7 (ATCC, TIB71) were grown on DMEM tissue culture medium supplemented with 10% heat-inactivated foetal calf serum (FCS) and Hepes 10 mM at 37°C in an humidified 5% CO_2_ atmosphere. For bacterial infection, cells were seeded in 24-well tissue culture plates 15 h before the experiment at a density of 7×10^5^ cells per well. Overnight cultures of *Y. enterocolitica* strains grown at 21°C were diluted 1∶10 into 5 ml of LB and grown with aeration at 37°C or 21°C for 3 h. Bacteria were pelleted, washed once with PBS and resuspended to an OD_600_ = 1 (approximately 10^9^ CFU/ml) in PBS. Cells were infected with this suspension to get a multiplicity of infection of 25∶1. To synchronize infection, plates were centrifuged at 200×*g* during 5 min. After a 30 min infection, cells were washed twice with PBS and then incubated for an additional 180 min in medium containing gentamicin (100 µg/ml). Supernatants were removed from the wells, cell debris removed by centrifugation, and samples were frozen at −80°C. TNFα present in supernatants of culture cells was determined by ELISA (Bender MedSystems) with a sensitivity <4 pg/ml.

### Statistical analysis

The results were analyzed by the one-sample *t* test using GraphPad Prism software (GraphPad Software Inc.). Results are given as means ± SD. A *P* value of <0.05 was considered to be statistically significant.

## Supporting Information

Figure S1 **Sequence alignment of StLpxR and YeLpxR.** Conserved residues are shown with black background. The amino acids that were mutated in this study are highlighted with grey background, with the two biggest differences, G/D31 and A/Q35, indicated by grey stars. The secondary structure for StLpxR is shown on top of the alignment.
(TIF)

Figure S2 **Analysis of the expression of** ***Y. enterocolitica lpxR*** **.** Analysis of *lpxR* mRNA levels assessed by RT-qPCR. Total RNA was extracted from YeO8 (white bar), and mutants (grays bars) YeO8-Δ*phoPQ* (Δ*phoPQ*), YeO8-Δ*pmrAB* (Δ*pmrAB*and YeO8-Δ*phoPQ*-Δ*pmrAB* (Δ*phoPQ-pmrAB*), Yvm927 (Δ*rovA*), Yvm927-Δ*phoPQ*-Δ*pmrAB* (Δ*rovA*Δ*phoPQ-*Δ*pmrAB*). (A) Bacteria were grown at 21°C. Wild-type bacteria (YeO8) expression levels were set to 1. (B) Bacteria were grown at 37°C. Wild-type bacteria (YeO8) expression levels were set to 1.
(TIF)

Figure S3 **Analysis of LpxR levels.** Cell envelopes were purified from YeO8-Δ*lpxR*Km mutant (YeO8-Δ*lpxR*) carrying plasmids pTMLpxRFLAG, pTMLpxR(N9A)FLAG or pTMLpxR(S34A)FLAG. Strains were grown at 21°C. 80 µg of proteins were run in SDS-12% polyacrylamide gel, electrotransferred onto a nitrocellulose membrane, and developed by using anti-Flag antibodies.
(TIF)

Table S1 **Primers used in this study.**
(DOC)
