# Supplementary figures and images for: Deciphering the Acylation Pattern of Yersinia enterocolitica Lipid A
Source: PLoS Pathog. 2012 Oct 25;8(10):e1002978. doi: 10.1371/journal.ppat.1002978 (PMC3486919; doi:10.1371/journal.ppat.1002978)

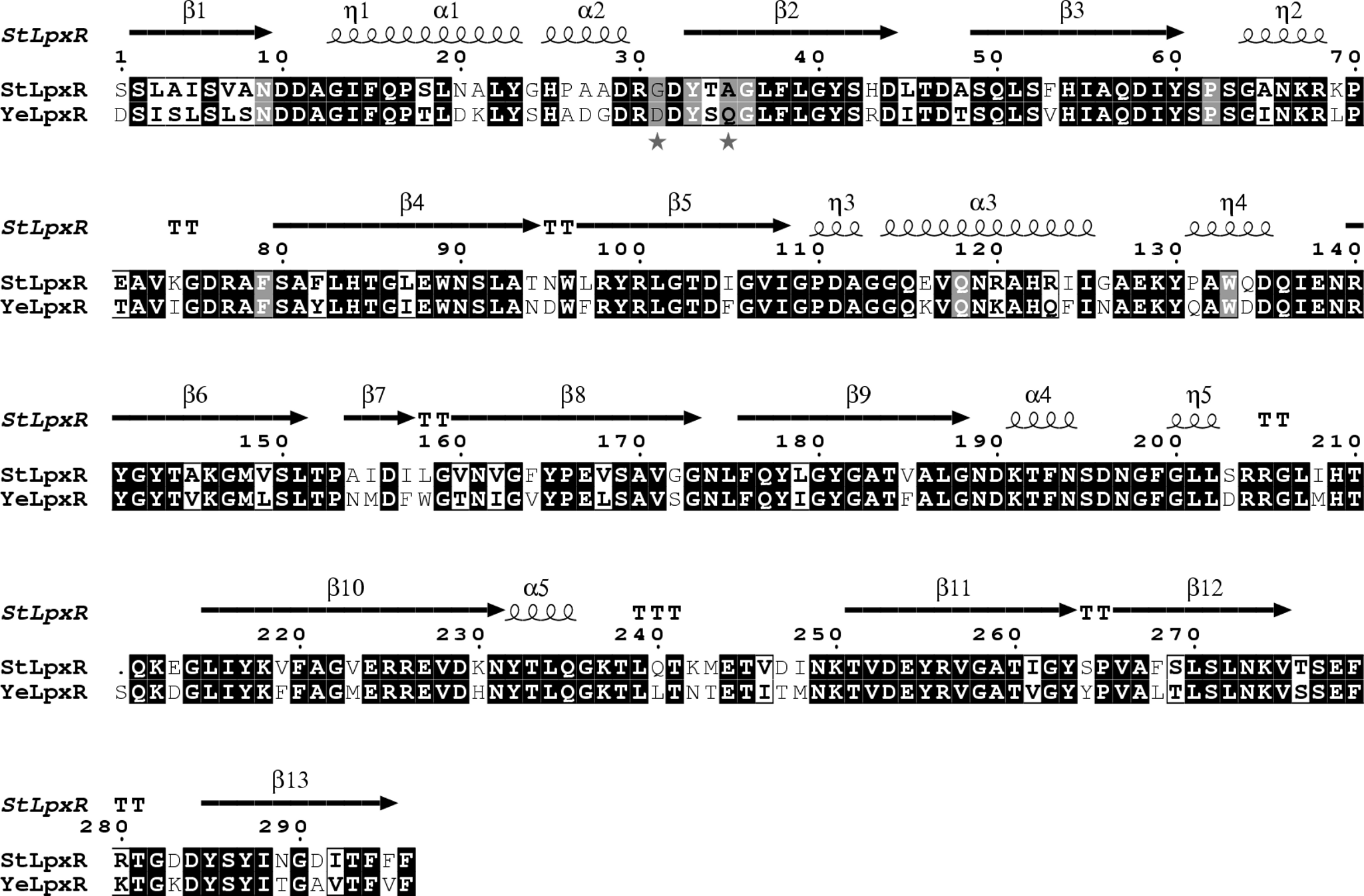

Supplement: Figure S1 — Sequence alignment of StLpxR and YeLpxR. Conserved residues are shown with black background. The amino acids that were mutated in this study are highlighted with grey background, with the two biggest differences, G/D31 and A/Q35, indicated by grey stars. The secondary structure for StLpxR is shown on top of the alignment. (TIF) [file ppat.1002978.s001.tif]

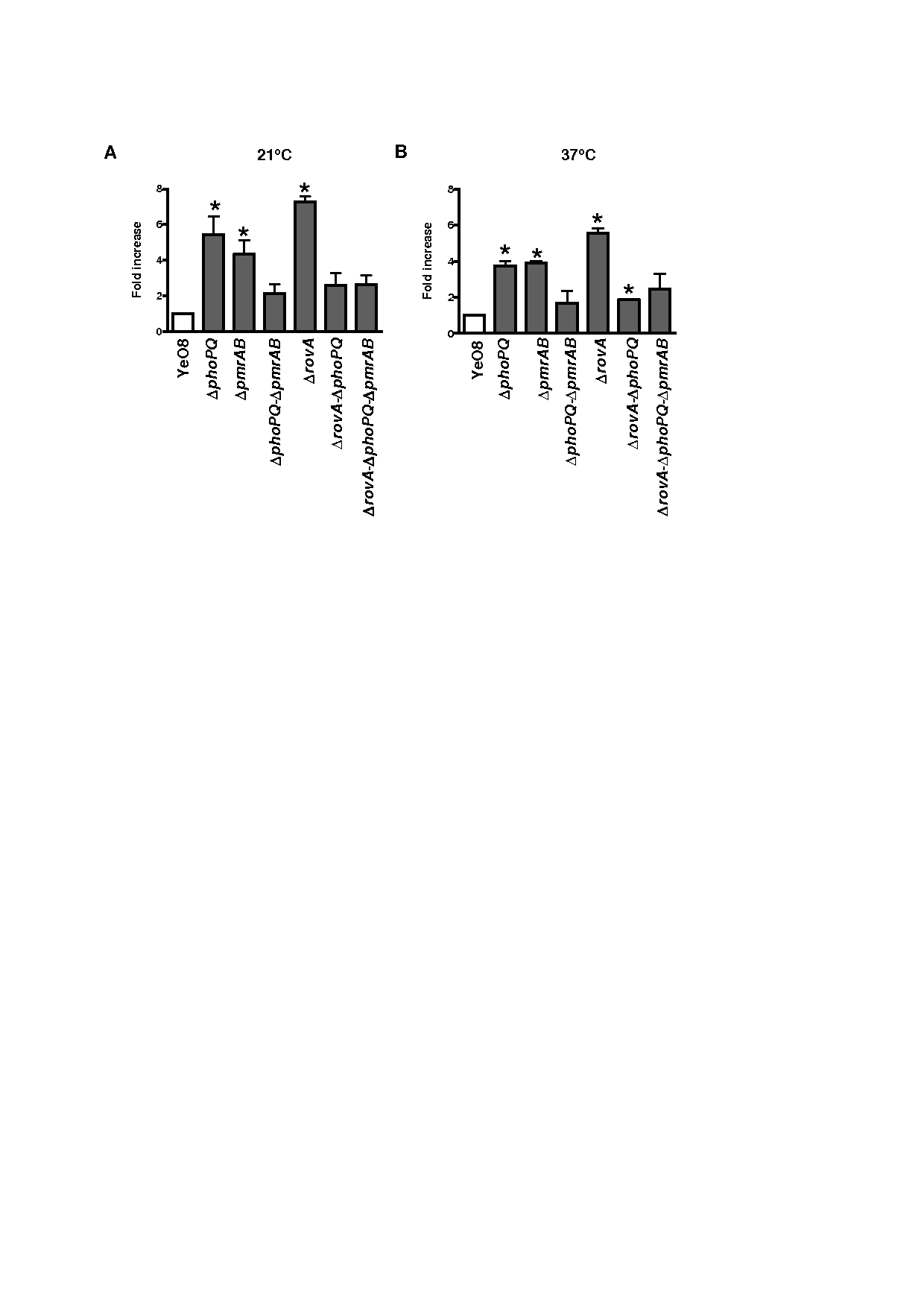

Supplement: Figure S2 — Analysis of the expression of Y. enterocolitica lpxR . Analysis of lpxR mRNA levels assessed by RT-qPCR. Total RNA was extracted from YeO8 (white bar), and mutants (grays bars) YeO8-ΔphoPQ (ΔphoPQ), YeO8-ΔpmrAB (ΔpmrABand YeO8-ΔphoPQ-ΔpmrAB (ΔphoPQ-pmrAB), Yvm927 (ΔrovA), Yvm927-ΔphoPQ-ΔpmrAB (ΔrovAΔphoPQ-ΔpmrAB). (A) Bacteria were grown at 21°C. Wild-type bacteria (YeO8) expression levels were set to 1. (B) Bacteria were grown at 37°C. Wild-type bacteria (YeO8) expression levels were set to 1. (TIF) [file ppat.1002978.s002.tif]

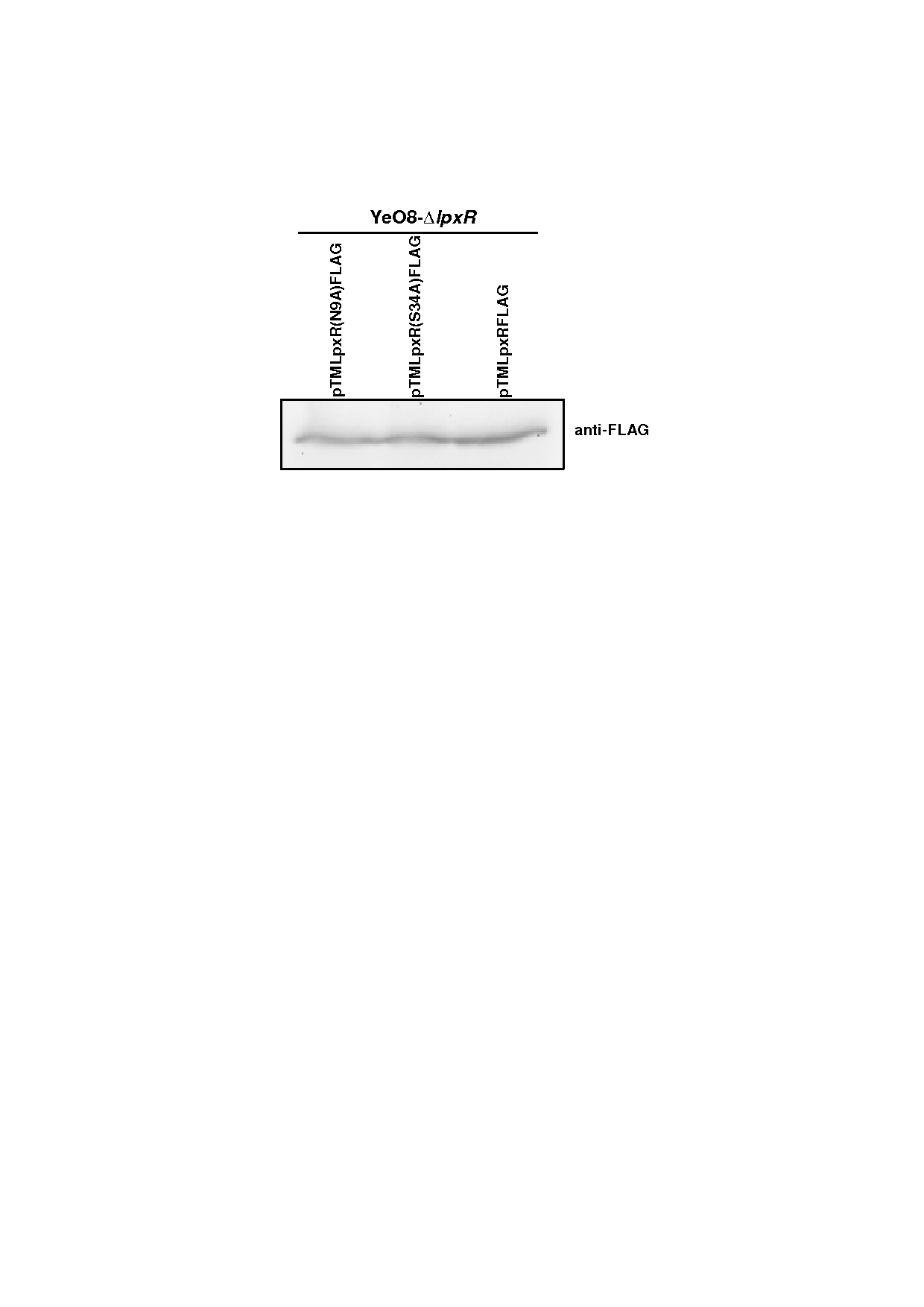

Supplement: Figure S3 — Analysis of LpxR levels. Cell envelopes were purified from YeO8-ΔlpxRKm mutant (YeO8-ΔlpxR) carrying plasmids pTMLpxRFLAG, pTMLpxR(N9A)FLAG or pTMLpxR(S34A)FLAG. Strains were grown at 21°C. 80 µg of proteins were run in SDS-12% polyacrylamide gel, electrotransferred onto a nitrocellulose membrane, and developed by using anti-Flag antibodies. (TIF) [file ppat.1002978.s003.tif]
