# Supplementary material for: Deciphering the Acylation Pattern of Yersinia enterocolitica Lipid A
Source: PLoS Pathog. 2012 Oct 25;8(10):e1002978. doi: 10.1371/journal.ppat.1002978 (PMC3486919; doi:10.1371/journal.ppat.1002978)
Supplement: Table S1 — Primers used in this study. (DOC) [file ppat.1002978.s004.doc]

**Table S1. Primers** used in this study.

| **Purpose** | **Name** | **Sequence (5' to 3')** |
| --- | --- | --- |
| **Mutagenesis** |  |  |
| *lpxR* | LpxRupF | CCTGAATTTAAAACCCCGAATG |
|  | LpxRupR | CGGGATCCCGGCGGTTGAAGTATCGGATTGC |
|  | LpxRdownF | CGGGATCCCGACAAGATATTTACTCCCCATCAGG |
|  | LpxRdownR | AATTCGATTCTTATTCTCGCCAC |
| *yopP* | YopPUpF | AGCTGACAACCATCGCCGAG |
|  | YopPUPR | cggatccggactcactatagggTCAGGATATTTATTGTTCGCCTG |
|  | YopPDownF | cggatccggactcactatagggGGTGAAAATCCTTTACCCCACG |
|  | YopPDownR | GGGGAAAGAACTGGCGATGC |
| *yopE* | YopEUpF | GCGAAATCTGATATTGCTGGCAC |
|  | YopEUPR | cggatccggactcactatagggGCTGTGAGACTGAGCGCCCAG |
|  | YopEDownF | cggatccggactcactatagggCGCCACGGGCAGCGGCCCTC |
|  | YopEDownR | ATATCATGTGCATGAAAGCCTGC |
| ***lpxR* site-directed mutagenesis** |  |  |
| N9 | MutN9AR | TGCTGATAAGGATAACGAAATACTACTATCAGC |
|  | MutN9AF | GATGATGCAGGTATTTTTCAACCAACAT |
| D10 | MUTD10AR | TTATGATAAGGATAACGAAATACTATCAGC |
|  | MUTD10AF | GCAGATGCAGGTATTTTTCAACCAACAT |
| D31 | lpxR D31AinvR | TGCACGATCTCCATCTGCATGACTATAT |
|  | lpxR D31AinvF | GATTATTCTCAGGGACTATTCTTAGGTT |
| Y130 | MUTY33AR | TGCATCATCACGATCTCCATCTGCATGAC |
|  | MUTY33AF | TCTCAGGGACTATTCTTAGGTTATAGTCGAGAT |
| S34 | MUTS34AR | TGCATAATCATCACGATCTCCATCTGCATGAC |
|  | MUTS34AF | CAGGGACTATTCTTAGGTTATAGTCGAGAT |
| G36 | MUTG36AR | TGCCTGAGAATAATCATCACGATCTCCAT |
|  | MUTG36AF | CTATTCTTAGGTTATAGTCGAGATATT |
| Q57 | MUTQ57AR | TGCTGCTATATGAACAGATAGTTGACTTG |
|  | MUTQ57AF | GATATTTACTCCCCATCAGGTATTAATAAAAGAC |
| P62 | MUTP62AR | TGCGGAGTAAATATCTTGTGCTATATGAAC |
|  | MUTP62AF | TCAGGTATTAATAAAAGACTACCAACTGCTGTAATAGGT |
| F79 | MUTF79AR | TGCAGCCCTGTCACCTATTACAGCAG |
|  | MUTF79AF | AGTGCATATCTGCACACAGGAATTGAATGGAACTC |
| Q118 | MUTQ118AR | TGCGACTTTTTGACCTCCAGCATCAGG |
|  | MUTQ118AF | AACAAAGCCCATCAATTTATTAATGCCGAAA |
| H122 | MUTH122AR | TGCGGCTTTGTT TTGGACTTTTTGACCTCC |
|  | MUTH122AF | CAATTTATTAATGCCGAAAAGTATCAAGC |
| W133 | MUTW133A | TGCTGCTTGATACTTTTCGGCATTAATAAATTG |
|  | MUTW133AF | GATGACCAAATCGAGAACCGCTACGGCTACACTGTT |
| ***lpxR* sequencing** |  |  |
|  | SEQMUTLPXRF1 | TATCGCATTATACGGCGCTC |
|  | SEQLPXRR1 | GCAGATATGCACTAAAAGCCCTG |
|  | SEQMUTLPXRF2 | CGCATCATCTATAGCCAAAGCTG |
|  | SEQMUTLPXRR2 | CCAGAAATCCATATTTGGGGTG |
| **Complementation** |  |  |
| *lpxR* | GeneLpxRF | AATCACGTTTTGGCCGCAAC |
|  | GeneLpxRR | GCATTAATAAATTGATGGGCTTTG |
| **Tagging** |  |  |
| *lpxR* | lpxRFtagging | AGAAATCAGTAGATGCGGATAAGC |
|  | lpxRRFLAG | gactacaaagatgacgacgagaaaacaaatgttacagctcctgttatgta |
| **Promoter region** |  |  |
| *lpxR* | PROlpxRR | AAACCTTATAAGCTCTCCGACG |
|  | PROLpxRF | GGAATTCCATCAGACTGAGGGCGGGATG |
| **RT-qPCR** |  |  |
| *lpxR* |  | ACAAGATATTTACTCCCCATCAGG |
|  |  | CAGATTACCACTTACCGCAGACAG |
| *rpoB* |  | GTACGCCGGTAGACATCGTT |
|  |  | TGGCAACTTCTTCCTGCTTT |
| *tonB* |  | AATGCACCGGTTAAATCAGC |
|  |  | CCTGTTTCACTTCACGCTCA |
